# Finding the Prevalence of Autism in Female Mental Illness: Improving Child Development for an Underdiagnosed and Undertreated Population

**DOI:** 10.3390/children12121600

**Published:** 2025-11-24

**Authors:** Robert McCrossin

**Affiliations:** Cooroy Family Practice, Cooroy 4563, Australia; bobmccrossin@gmail.com

**Keywords:** autistic, female, prevalence, overshadowing, adolescent, perinatal, mental, suicide, Pareto, male

## Abstract

Structure of the study: *Aims*: The primary aim is to explore intergenerational clinical issues caused by the underdiagnosis of female autistic spectrum disorder (ASD) in mental illness (MI) patients by calculating the proportion of patients with mental health conditions who are autistic. Secondary aims are to derive further values for the true prevalence of female ASD and to derive a mathematical model to estimate the improved efficiency of management based on the correct diagnostic formulation. *Context*: Review diagnosis problems and background issues relating to female autism which affect the diagnosis and management of ASD and associated MIs. *Methodology*: An inductive process using Bayes’ theorem including a novel form akin to a medical test with secondary data from peer-reviewed sources, and the key variable of the unbiased value for the prevalence of ASD in females. Derivation of a model for management efficiency based on the Pareto Principle. *Results*: Prevalence values for ASD in various mental illnesses and conditions consequent on or associated with ASD and MI. Further data for the prevalence of female ASD with a range of 19 values. Estimation of the efficiency gains as advocacy for the revision of methods of treatment. *Discussion*: The centrality of diagnosing ASD in mothers with mental illness, in particular perinatal depression, to break a common intergenerational cycle. Problems to overcome and aspects of effective management including environmental and therapeutic interventions. *Summary*: This paper will, for the first time, calculate the proportions of children and young women with a mental illness (MI) who are autistic, and consider the consequences. Recent information suggests female autistic spectrum disorder (ASD) is much more common than previously thought, with a likely prevalence of 6% and with 80% undiagnosed at the age of 18. ASD then becomes a common comorbidity of female mental illness with nearly one in five women who develop a mental illness being autistic. ASD has heretofore been regarded as a pediatric condition and, though now thought to be lifelong, it is still not well recognized by adult health services. Most mental illness first presents in the teens and early twenties, although anxiety can begin even earlier. Comorbid ASD is more difficult to diagnose due to diagnostic overshadowing, and ASD comorbidity makes the mental illness more severe and more difficult to treat. The consequences of perinatal depression are particularly concerning due to their intergenerational effects. Recognized ASD is readily treatable with an approach empathetic to neurodiversity. Improving the transition from adolescence to young adulthood by increasing knowledge of autism in adult health services would dramatically improve female mental health at surprisingly little effort or extra cost.

## 1. Introduction

### 1.1. Problem Statement

It is common for autistic females to have an associated mental illness (MI) (Section 1.7). Females with autistic spectrum disorder (ASD) commonly camouflage, or mask, their condition (Section 1.3) and therefore, if they present with ASD at all, they first present with their mental illness or illnesses. As a result, there are many studies of the prevalence of MI in diagnosed autistic women, but very few of the prevalence of ASD in women with an MI. MI is more difficult to manage in the presence of ASD (Appendix B.1), so diagnosing ASD is critical for effective management. Key to finding the extent of the problem is the value of the overall prevalence of female ASD, which is used to calculate the proportion of females with ASD in MI. This paper will present information that suggests that the overall prevalence of both ASD in females and the prevalence of ASD in female MI are both far higher than generally thought, and, for the first time, calculate the proportion of young women with a number of MIs who are autistic, and consider the consequences for management over the intergenerational cycle. Identity first language is preferred in this paper.

### 1.2. The Importance of Autism Spectrum Disorder (ASD) as a Mental Health Comorbidity in Adolescent Girls and Young Women

It is self-evident that, to manage ASD, you have to know it is present. If female ASD is not diagnosed prior to transitioning to adult care, it is likely that the diagnosis will not be made (Section 1.7), especially in the presence of MI. This will impact the entire intergenerational cycle, since maternal undiagnosed ASD affects the prevalence and severity of perinatal depression, which in turn impacts the severity of mental health and associated conditions in her offspring, and, in particular, in her daughters in the next generation.

In 2024, Cage et al. [1] surveyed 225 adults with autism, of whom 50 had children with autism. The adults with autism ranked 25 research topics in order of importance to them. The rankings included the following:

First: Mental health conditions and mental wellbeing.
Second: Identifying people with autism/diagnosis.
Fifth: Issues impacting women with autism.

This paper will address the relationships between these three topics. The primary task is to calculate, for the first time, the proportion of young females with mental illnesses who have comorbid ASD. It will show why this proportion is unknown, how to calculate the proportions, why this is important to know, and then discuss ways to improve diagnosis, therapy, and service delivery for these girls and young women.

ASD is a neurodevelopmental disorder with two major domains: persistent impairment in reciprocal social communication and social interaction, and restricted, repetitive patterns of behavior, interests, or activities. This latter domain includes hyper- or hyporeactivity to sensory input. It is defined in *The Diagnostic and Statistical Manual of Mental Disorders*, 5th edition, text revision 2022 [2]. The first version (DSM-5) was published in 2013. It provides a deficit model which expresses ASD behaviors as disorders compared to normal behaviors. Since that time, there has been growing interest in an alternate philosophical model emphasizing difference rather than deficit. This model of neurodiversity, where the neurodivergent differs from the neurotypical, is outlined in Section 1.5 and is the model favored in this paper.

Childhood autism was first described in 1926 in Russia by Grunya Sukhareva in the case studies of six boys [3]. In 1927, she reported the case studies of five girls [4]. These studies predated those of Kanner and Asperger (in boys only) by 17 years. Unfortunately, the first English translation of the female study was not published until 2020 [5]. This delay of 93 years has not been helpful in delineating the features of autistic females. Sukhareva initially described the condition as schizoid psychopathy but used the term autistic to describe certain behaviors. In 1959, she adopted the term autistic (pathological avoidant) psychopathy, anticipating pathological demand avoidance, which 66 years later is still not a term used in the DSM-5-TR or the International Classification of Diseases 11. She was a skilled observer, and described the sensory issues not included in the DSM until DSM-5 was published in 2013. She recognized that the condition encompassed children of normal intelligence and high achievers. Her observations were very modern and essentially matched the current female phenotype described in the DSM-5-TR:

“girls without intellectual impairments or language delays may go unrecognized, perhaps because of subtler manifestation of social and communication difficulties. In comparison with males with ASD, females may have better reciprocal conversation, and be more likely to share interests, to integrate verbal and non-verbal behavior, and to modify their behavior by situation, despite having similar social understanding difficulties as males. Attempting to hide or mask autistic behavior (e.g., by copying the dress, voice and manner of socially successful women) may result in the underdiagnosis of ASD in some females. Repetitive behaviors may be somewhat less evident in females than in males, on average, and special interests may have a more social (e.g., a singer, an actor) or ‘normative’ focus (e.g., horses), while remaining unusual in their intensity”.

Masking (or camouflaging) is very common and is discussed in Section 1.3. It appears to begin very early, and there must be environmental factors with networked causal pathways for camouflaging and other factors which might include the fact that girls are [6]

1. “Girly” in their interests and the subtleties of approach to their interests are missed;
2. Less likely to externalize and more likely to be anxious;
3. Inherently more socially aware;
4. Better in their apparent language skills, at least initially;
5. Better at masking during tests;
6. Disadvantaged by tests tailored more for boys.

There is extensive qualitative information that female ASD is considerably more common than current estimates (Section 1.3). Recent quantitative information suggests that it is at least as common as in males [7] and possibly even more common over the life span [6]. There has only been one published numerical estimate of female ASD prevalence allowing fully for biases in recognition and diagnosis [6]. If this estimate of 6% is valid, then clinical reasoning about female ASD needs a new frame of reference. ASD may be comorbid with most mental health conditions [8], and this is not well recognized by mental health services (Section 1.7). If the ASD diagnosis is made, 75% are first diagnosed with another condition and the ASD diagnosis may be delayed by up to 8 years [9]. This makes these conditions more difficult to manage in psychosis [10], anorexia nervosa [11,12,13,14], and anxiety and depression [15,16], with an increased risk of suicide [17]. It is well documented that therapy in ASD has to be tailored to the neurodivergent mind, or it will not succeed (Section 4.4). This paper aims to use Bayes’ theorem [18] to calculate the proportion of females with a MI who have comorbid ASD. This derivation crucially requires the true value of the prevalence of ASD in the female population, designated P(ASD). The outcome sought is to demonstrate the clinical importance of ASD in female MI and the necessity to increase the rate of ASD diagnosis. This would lead to better targeted therapy, improved access by increased efficiency of management, and consequent greater overall mental wellbeing. Currently, missed diagnosis and misdiagnosis cause a lot of unnecessary suffering, as this case report by Isaac et al. [19] demonstrates.

The format of the study is a population analogy of a clinical case: diagnosis, therapy, and prognosis. The paper is then an exercise in quantitative induction with subsequent risk management. The problem with testing a hypothesis by deductive reasoning is that, in an observational study, information supporting an alternative hypothesis tends to be discounted [20,21], particularly if alternative explanations are deemed to be rare. The starting point of this paper is the observation that female autism is not rare, and so its importance needs to be explored. This type of study is hypothesis-generating. The aim here is not a clinical diagnosis but an assessment of the clinical risk of the comorbid relationship for females with MI who are on the spectrum and who have not been diagnosed. The focus will be on cis-gender girls and young adult women. Specific problems for transgender individuals will not be examined. The reason is this complicates the definition and quantification of female MI. Transgender ASD with comorbid MI is an extremely important area, and likely fairly common. The range of data so far reported is wide and, with discordant sex and gender reporting, is more complex. It deserves its own study.

A much higher prevalence, or probability P(ASD), in the female population might show levels of ASD in MI that must be addressed. A key factor contributing to this problem is camouflaging, also described as masking.

### 1.3. Camouflaging

P(ASD) has generally been reported to be around 0.01, or 1% of the female population. There appears to be a female phenotype not well recognized [22], and a significant contributing feature is female camouflaging, now well described qualitatively [23,24] and with quantitative estimations of about 90% of ASD females obtained through histories [24,25] or by observation of its effect [6]. The effect observed by parents and teachers is excellent behavior in school or even preschool, with meltdowns between the classroom and home. The information on this behavior was gained via parental histories in a survey over a period of 3 months during routine diagnostic practice. The listed ages of the girls in Table 1 were at the time of the study, which sets an upper limit on the age of onset of the behavior. There are two peaks corresponding to the starting of school and the onset of puberty, where there is increased stress. The median age was 8 years and 4 months, ranging from 3 years and 4 months to 17 years and 4 months, with 15.9% starting under 6 years. Camouflaging clearly causes psychological distress from an early age.

**Figure 1:**
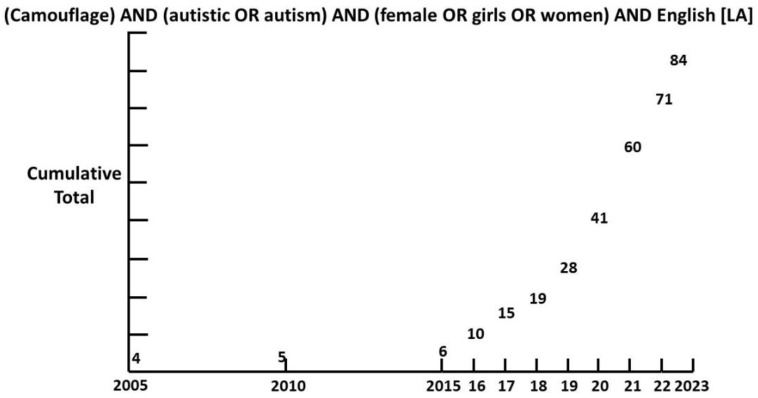
Cumulative total from a Pubmed search for papers on camouflaging on 26 June 2023.

Though camouflaging was first described by Wing in 1981 [26], it is only since 2015 that interest has grown exponentially (Figure 1).

With such a high proportion of individuals camouflaging, it is not surprising that a minority of females are recognized as possibly having autism [6], let alone diagnosed. The neurotypical world of the majority imposes high social expectations on female behavior. From a young age, autistic girls strive to fit in with their neurotypical peers, at great psychological cost [27]. The key to successful management is the acceptance of their neurodivergence, and the tragic irony is that they are so good at hiding it. A key aim of this paper is to reduce this effect by increasing the general knowledge of ASD and to try to turn a vicious circle into a virtuous one.

Camouflaging in ASD is part of a larger problem in female neuroscience. There is a large gender bias where, for example, only 0.5% of brain imaging articles have considered factors specific to women, such as postpartum depression [28].

### 1.4. Diagnostic Overshadowing

The underdiagnosis of ASD in the general female population is exacerbated when ASD is associated with an MI due to diagnostic overshadowing, or the misattribution of ASD features to the MI. This may hinder a patient from receiving an appropriate assessment because the assessor views her symptoms through the lens of another condition. The overshadowing can occur in the other direction, and a young autistic woman may be pejoratively labeled as “hysterical” [29]. Rather than the archetypical externalizing male presentation, female autism usually presents differently, with more internalizing symptoms [30] and “girly” interests [6]. Of particular relevance when considering the challenges faced by females with ASD is the concept of neurodiversity.

### 1.5. Neurodiversity: The Set of the Neurotypical and the Neurodivergent

“Who in the world am I? Ah, *that’s* the great puzzle!”.

Alice, from *Alice’s Adventures in Wonderland* by Lewis Carroll [31].

The basic premise of neurodiversity as expressed by Singer [32] is that there is a range of differences in individual brain function and behavior traits. Those who diverge from the societal norm are not necessarily disabled. While some may be disabled, as within the neurotypical majority as well, the problem is that the dominant societal paradigm does not suit neurodivergent people. The scope of neurodivergence is a matter of active debate [33], but there is no disagreement that autism is central to the concept. The root cause of most of the problems is a lack of mutual understanding, characterized as the double empathy problem [34]. The implications for management are simple but profound. Neurotypicals, both therapists and contacts of all degrees of intimacy, need to understand the mutual misunderstanding and allow for it. Neurodivergent individuals need to do the same, but, as “aliens on Earth”, have to make extra adjustments. Society as a whole is not going to readily understand, and the neurodivergent are often going to have to adjust. Practical advice might be to learn to camouflage well when necessary but learn strategies to minimize the pressure. Then make your excuses, leave, and become again your authentic self.

The therapeutic interaction should be identity-affirming [35]. It is important to acknowledge that identity is a key component of mental health treatment. The act of validating symptoms and experiences, allowing accommodations when requested, and exploring identity formation regardless of diagnosis, allows all patients who identify as neurodivergent to benefit from treatment. The aim of therapy is not to “cure” the individual. Autistic spectrum conditions (ASCs) and ASD can usefully be seen as different, with the disorder as a subset of the condition [6]. The aim is to minimize the disorder element and manage in society. An ASC is permanent and not a pathology, but an individual with an ASC who has significant problems coping in a pervasive neurotypical environment, with or without a comorbid illness, has a disorder and requires assistance to cope successfully. Obviously, understanding that ASD is present is required in order to manage it and professional assistance is required for comorbid MI. About 80% of autistic females have a comorbid mental health condition (Section 3.1.12). The comorbid illness often presents during the period of transition to adult care (Section 3.1), and the transition is often poorly managed [36,37]. The pathological element of ASD usually has a comorbidity as the major driver [38], and the analysis of this relation is a major theme of this paper.

### 1.6. ASD and Attention Deficit Hyperactivity Disorder (ADHD)

Within neurodivergence there is generally believed to be a polythetic taxon, which is a shared pattern of features that need neither to be universal nor constant among members of a class. The class includes ASD and ADHD. There is a continuous field of clinical characteristics which are best managed without worrying about a precise categorical diagnosis. A categorical diagnosis is fraught with danger, since factors contributing to a behavior not consistent with the chosen category may be misinterpreted or overlooked. Neurodivergence is part of the differential diagnosis of a quiet girl failing academically at the back of the class. She may be camouflaging her autistic communication style to avoid standing out. She might have been bullied by classmates or shamed by the teacher when she tried to participate. She might not be able to focus or concentrate, especially if she has no interest in the subject. She may have an auditory processing condition and not be able to follow the teacher. She may be very good at deliberately processing information, but the teacher may be skimming the topic or switching subjects too often. She might be very sensitive to noise and too distracted to pay attention. She may be comorbidly highly anxious. She just hates being in that class or that school. To tease out the multilayered elements requires carefully listening to her in a non-threatening environment. Management will require a multidisciplinary approach involving environmental adjustment, education in mutual communication between the neurodivergent and neurotypical, talking therapies, and medication, tailored to her individual circumstances. She must be comfortable with the program. For the management of autism in particular, a top-down approach will not work [18]. Any distinction between ASD and ADHD is complicated by many ADHD features also being explained by features of ASD [39]. Since ADHD, like ASD, is not necessarily seen as a mental illness, a rate of comorbidity will not be considered in this paper. The possibility of cryptic ASD should always be considered when ADHD is diagnosed and vice versa. The term AuDHD for the combination is now being used within the neurodivergent community and by peer-reviewed papers, but it is not yet a formal diagnosis. The two are so intertwined that if ADHD and an MI comorbidity are not responding to therapy, ASD should be considered. The synergy in AuDHD appears to cause a high risk of behavioral, psychiatric, and clinical conditions [40] including substance abuse, accidents, and offending behavior [41]. My own clinical experience suggests that anxiety and oppositional defiant disorder are particularly relevant features. While the overlap of ADHD and ASD may create diagnostic confusion, the problem is much wider and extends to most areas of MI.

### 1.7. Mental Illness Guidelines and ASD

On the adult side of the pediatric/adult clinical divide, mental health services do not appear to have fully appreciated the potential importance of ASD as a comorbidity in MI, especially in females. This may also apply to transition guidelines. There are a growing number of papers considering this relationship, but these are generally coming from an ASD focus and invariably conclude that adult mental health services need to pay more attention to ASD. The lack of diffusion across the barrier is likely due to not appreciating either the frequency or the severity of the interaction between ASD and MI. The product of frequency and severity is the way in which risk is quantified. The literature on the severity of the relation again comes from an ASD focus, and the lack of a quantified P(ASD), until recently, together with female camouflage, have hidden the frequency. We will examine a selection of recent papers and guidelines as examples that demonstrate aspects of the problem.

1. The National Institute for Health and Care Excellence (NICE) guideline in the 2022 [42] entry for the treatment and management of depression in adults lists bipolar disorder, PTSD, and anxiety as comorbidities. If there are “language or communication difficulties e.g., autism”, the guideline suggests referring to the NICE Autism guideline but does not mention female camouflage possibly hiding comorbid ASD. The assumption appears to be that the possibility of ASD has been recognized. If it has not, the skills of female camouflaging would require a very good understanding of female ASD by the clinician. The language and communication skills in female ASD can appear quite satisfactory, and the pathway would be difficult to navigate for a general practitioner/primary care physician.
2. An adolescent depression primary care screening in 2018 did not mention ASD among mental health risk factors [43].
3. A study of treatment-resistant depression in primary care in 2018 [44] did not mention the possibility of comorbid ASD, though it did generate a response which did so [45].
4. A 2021 paper on postpartum depression and psychosis [46] examined the prevalence of neurodevelopmental disorders in children but not in mothers.
5. It has been suggested [47] that pediatricians have a role to play in reducing perinatal mortality and noted that some do screen for maternal depression. ASD is not mentioned. We would add that this is an area where pediatric expertise could indeed be very useful if looking for ASD became routine, perhaps in partnership with midwives [48] aware of both depression and ASD (Section 3.1.6 and Section 4.5).
6. A study [49] characterizing treatment-resistant anorexia nervosa (TRAN) from 2000 to 2016 in patients aged 17 and upwards was published in 2021. It did not mention ASD but did speculate that TRAN might be a different concept warranting additional research. There is qualitative information to support this [36,50].
7. The American Psychiatric Association 2023 guideline [51] for treating eating disorders lists other psychiatric disorders that should be particularly considered. ASD is not among them.
8. A recent review of eating disorders [52] does not mention the relation of ASD to anorexia nervosa (AN) though it does mention the relation to avoidant–restrictive food intake disorder (ARFID), discussed in Section 3.2.5.
9. A 2023 paper [53] on difficult-to-treat bipolar disorder mentioned ADHD, but not ASD.
10. A discussion of treatment resistance in mental illness [54] lists autism being misdiagnosed as schizophrenia but does not list schizophrenia spectrum disorder as being comorbid with ASD.
11. A 2023 paper [55] listing comorbidities of borderline personality disorder (BPD) quotes BPD as a comorbidity in 37.7% of ADHD cases but does not list ASD.

The obvious conclusion is that knowledge possessed by ASD-focused practitioners, generally but not solely on the pediatric side of the divide, is not reaching our adult colleagues. If indeed the risk of undiagnosed ASD is much higher than currently believed, it becomes essential to manage it. It is to the elements of this risk assessment that we will now turn.

### 1.8. Problems Diagnosing the Prevalence of Female ASD

**Figure 2:**
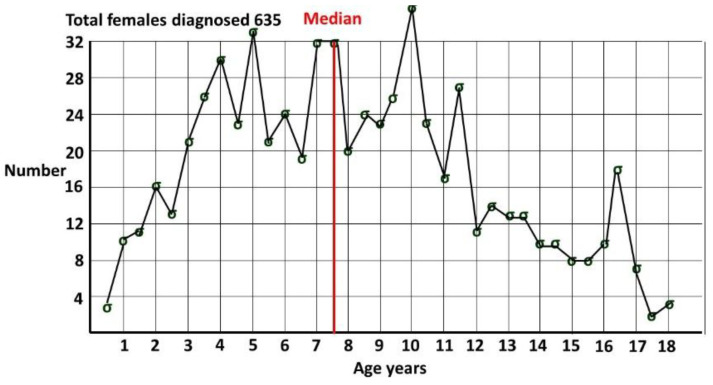
Age at diagnosis of female ASD in a community-referred pediatric clinic.

We can only rely on a measurement of female ASD prevalence P(ASD) if we are sure that all ASD patients in the measured population of interest have been recognized and diagnosed. A lack of initial recognition appears to be the major problem rather than a diagnostic bias [6]. Community professionals do miss girls in diagnosis [6] but at a fairly low rate compared to research studies. In my referred community specialist clinic [6] (1052 males and 659 females), the male-to-female odds ratio (MFOR) was 1.596. A recent study [56] showed a “leaky pipeline” in the comparison of the MFOR for the community versus research diagnosis of females. The research standard was the Autism Diagnostic Observation Schedule (ADOS) module 4. The MFOR for the community databases of referred patients (9192 males and 5768 females) was 1.594. The MFOR for the research ADOS assessments (189 males and 25 females) was 7.56. The ADOS relies on an observation protocol, and it is difficult not to believe that camouflage was a serious confounding factor. The Centers for Disease Control (CDC) prevalence [57] for ASD at 8 years of age is often incorrectly referenced as the prevalence of ASD. The age qualification is often omitted when cited. In any cohort, the overall prevalence must be greater as cases accumulate after this age, as demonstrated in Figure 2 from my pediatric clinic, showing females diagnosed by me [6], all with a known date of diagnosis. Females were defined by sex of assignation at the time of diagnosis. The median age of diagnosis was 7 years and 6 months. At 8 years, only around half of the pediatric-age diagnoses in females have been made.

### 1.9. Conditional Probability and Bayes’ Theorem

**Figure 3:**
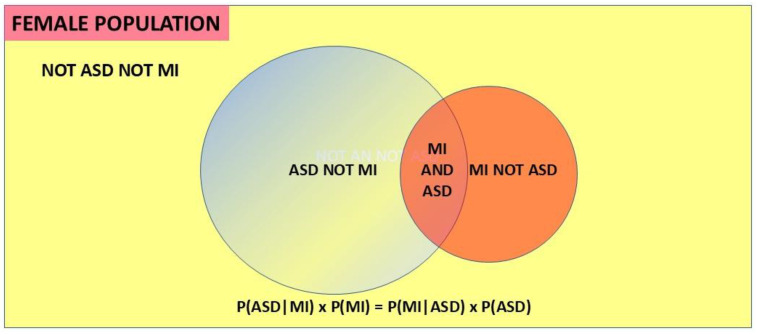
Venn diagram of Bayes’ theorem showing the interacting populations.

Bayes’ theorem is a statistical method of taking initial (prior) information and using further information to derive refined information. The process of clinical diagnosis is inherently Bayesian [58]. Further information is sequentially added to a provisional diagnosis until the probability of the refined diagnosis is high enough to act upon. It is often the only way to reach certain conclusions in a timely manner. This is the case in this study. The process of refining basic data to derive a conclusion is induction and is the bedrock of the scientific process. Since induction uses available data to solve an unknown, it is well placed to find and quantify the presence of an overshadowed condition when it is being overshadowed. Due to the problems outlined in Section 1.8, it would take many years to establish the proportion of ASD in female mental illnesses by direct measurement in those populations. Due to recent developments by the author of a new application of Bayes’ theorem [18] and the calculation of an unbiased value of 6.0% for the population prevalence of female ASD [6,18], these proportions can be calculated using current published data, and their clinical importance assessed. There are four prevalence variables to consider. A prevalence is mathematically a proportion, or a probability. The first two variables are the female population prevalence of ASD, P(ASD), and of MI, P(MI). These are called base rates. The other two variables are conditional probabilities. When a proportion of population ASD interacts with the total population of MI, MI is designated the base and the interacting proportion of the population of ASD is designated as the probability of ASD *given* MI, or P(ASD|MI). Obviously then a proportion of population MI must interact with the total population ASD, and then ASD is the base and the interacting proportion of population MI is designated as P(MI|ASD). This is shown in the Venn diagram (Figure 3). P(ASD|MI) is the variable we need to find. The probability of ASD in a population of MI can be found by using versions of Bayes’ theorem.

The simplest form of Bayes’ theorem is firstly the probability form

P(ASD|MI) × P(MI) = P(MI|ASD) × P(ASD)(1)

Secondly, another version used in medical diagnoses is the odds form

Odds (ASD|MI) = likelihood ratio × Odds (ASD)

Probability P and odds O are interconverted by the formulae

Odds O = P/(1 − P) and P = O/(1 + O)

The likelihood ratio (LR) is P(MI|ASD)/P(MI|not ASD). Here the MI is being used as a test for ASD, and LR is the ratio of the true positive rate and the false positive rate for the test. Values may be reported for the prevalence of MI in a population subset with ASD, P(MI|ASD), and the prevalence of MI in the complementary part of the population without ASD, P(MI|not ASD). The ratio of P(MI|ASD) and P(MI|not ASD) is called the risk ratio or relative risk (RR). It is mathematically equivalent to the likelihood ratio and can be used as such. Hazard ratios (HRs) are often reported in the literature and are mathematically equivalent to likelihood ratios [18], though the derivation and purpose are different. Hazard ratios describe the relation at a particular instant of time, and this must be kept in mind when interpreting the result, since the rate of diagnosis of each condition can change with age. The details of this form of Bayes’ theorem are discussed in Appendix A, where a formula for P(ASD|MI) is derived as follows:

HR_MI_/(15.667 + HR_MI_) (2)

Both the probability and odds forms are used in the current paper to derive P(ASD|MI). The inverse forms of the ASD/MI relation present quite different practical problems for the clinician. Since female ASD is less visible than the comorbid MI.

MI *given* ASD: (MI|ASD), this is difficult. The combination is hard to manage because it is more complex than the MI alone. The therapist may lack the necessary skills, but knows ASD is present and can refer for expert help.

ASD *given* MI: (ASD|MI). This is much more difficult. The undiagnosed ASD with comorbid MI may be intractable to therapy and the therapist does not know why, and the person they refer to may not know either.

### 1.10. P(ASD)

The value of P(ASD) is the critical variable in both versions of Bayes’ theorem. The Bayesian relations hold if the unknown we are usually seeking, P(ASD|MI), is known and the unknown is P(ASD). P(ASD) was calculated [18] using a HR in a Danish population study [59] from 1981 to 2008. The data had quite different prevalences of anorexia nervosa (AN) and ASD to contemporary values, but the result of 0.060 was the median of the 19 values calculated to date (Section 3.3). These include the five calculated in this paper where the unknown variable was P(ASD). Finding an appropriate value for P(ASD) using outdated estimates of the variables with the HR method was quite unexpected, and the reason is demonstrated in Appendix A.

If the estimate of 0.060 used for the P(ASD|MI) calculations is indeed valid, then the proportion of ASD in female MI becomes of significant quantitative importance in diagnosis, therapy, and funding, and the risks can be estimated. The secondary data to which the formulae are applied, i.e., P(MI), P(MI|ASD), and HR_MI_, are all referenced published values.

## 2. Methods

To enhance readability for non-technical readers, all mathematical derivations and detailed notations have been moved to Appendix A and Appendix B. The main text provides a step-by-step, plain-language description of the procedure. Until a plausible estimate of P(ASD) allowing for the biases in estimation became available, it was not possible to use Bayes’ theorem to determine the proportion of ASD comorbid with overshadowing mental health conditions. Once this became available, it was also possible to design a method of assessing the efficiency of successful clinical intervention (here based on a Pareto analysis). Secondary data to support the Bayes’ and Pareto analyses have been patchy and incomplete, and the structure of the analyses will allow prospective studies to gather the data necessary to refine the estimates.

The target quantities include the following:

1. P(ASD|MI) using the probability form of Bayes’ theorem with P(ASD), P(MI), and P(MI|ASD).
  P(ASD|MI) using the odds form of Bayes’ theorem with P(ASD) and the RR or HR.
2. P(ASD) using the probability form of Bayes’ theorem if data is opportunistically available with P(MI), P(MI|ASD), and P(ASD|MI).
3. Estimates of savings in therapist time to allow for an improved overall patient care by Pareto analysis. The derivation of the Pareto formula and calculation of possible efficiencies are detailed in Appendix B.

In summary, we estimate how many girls and young women in mental health care are likely to be autistic. Using conditional probability (Bayes’ theorem), we combine known base rates with co-occurrence data. A Pareto analysis highlights which small set of measures explains most of the variation, guiding screening, service planning, and advocacy.

## 3. Results

### 3.1. P(ASD|MI) for Selected Mental Illnesses in Children, Adolescents, and Young Adults

#### 3.1.1. Context

**Figure 4:**
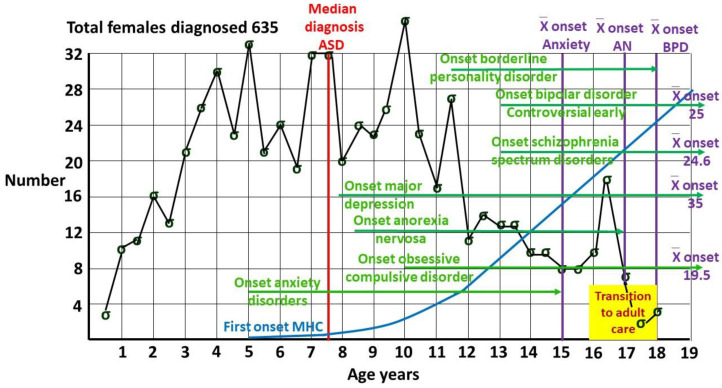
Indicative first and mean onset of mental illnesses in the pediatric-age group.

Figure 4 shows indicative timings of the onset of comorbid illnesses in females, putting the results in context. Half of all individuals with a mental health disorder have their onset by 18 years, and 62.5% by age 25 [60]. Mental illness is a problem for the young, emphasizing the importance of an informed transition to adult services.

We can now calculate the proportion of ASD given different mental illnesses. The range of values for P(MI|ASD) in a particular illness can be wide, and recent and reasonably conservative data have been used where available. There are in fact not very many published results, and precise values are not critical. Where variation can be quantified, it will be reported in the results. The Bayesian formulae use my value for P(ASD), and the other variables are referenced secondary sources from the literature. If a usable bound can be derived from these secondary sources, it will be reported in the results. Since the aim of the study is to demonstrate that the level of ASD in a mental illness is high enough to measure and act upon, variations that might reduce the value of P(ASD|MI) will be the focus. For P(ASD), the 1st quartile value is 0.055 (Section 3.3), which would reduce P(ASD|MI) to 91.7% of its measured value. A general possible reduction with the HR of ~15% will be discussed in Appendix A. The aim of this study is to set a new frame of reference to establish the clinical importance of the relationship of the cryptic ASD with comorbid MI. One might substitute different published values for P(MI|ASD) and P(MI) but very few are likely to deliver a level of risk for P(ASD|MI) below clinical concern.

#### 3.1.2. Depression (DP)

Kirsch et al. [61] gave a hazard ratio for depression-related diagnoses of 2.28, which by Formula (2) gives a P(ASD|DP) of 12.7%. The lower bound of the HR 95% confidence interval (CI), the 2.5th centile of 1.82, reduces the P(ASD|DP) to 79.8% of the value, i.e., 10.4%. Pezzimenti et al. [62] gave the P(DP|ASD) of 0.202 and P(DP) of 0.084 for adolescents. Formula (1) gives the P(ASD|DP) of 0.144 or 14.4%. Hudson [63] gave an effective lifetime hazard ratio of ~4, giving a P(ASD|DP) of 0.203 or 20.3%, consistent with the comorbidity with ASD leading to more prolonged, visible, or severe depression over time.

#### 3.1.3. Perinatal Depression

Pohl et al. [64] found a postnatal depression rate P(PND|ASD) in autistic women of 60%. This was potentially signaled by an antenatal rate of 40%. Luca et al., in a 2017 study [65], found a postnatal rate in pregnancy, P(PND), of 11.5%. Then, we find

P(ASD|PND) = 0.313

The likelihood of autism in postnatal depression may be a shocking 31.3%. Two-thirds of these mothers will have antenatal depression [64], and it is essential to make neurodivergence-friendly arrangements to ease the stress of birthing for autistic mothers [48].

#### 3.1.4. Anxiety Disorders (ANX)

Kirsch et al. [61] gave a hazard ratio for anxiety-related diagnoses of 2.91, which gives a P(ASD|ANX) of 15.7%, reduced when using the HR 2.5th centile of 2.28 to 12.7%. Croen et al. [66] gave data for anxiety-related diagnoses in adults, allowing the calculation of a hazard ratio of 3.22, which gives a P(ASD|ANX) of 0.170 or 17.0%.

#### 3.1.5. Social Anxiety Disorder (SA)

Values vary for P(SA|ASD) but are generally high. If we use an indicative value of 0.45 with a lifetime P(SA) of 0.103 [67] and with a P(ASD) of 0.060, we find a P(ASD|SA) of 0.262 or 26.2%. This is higher than the estimate for anxiety-related diagnoses, but this is not surprising given that social communication is the major problem in ASD. The overall conclusion is that ASD is common in anxiety disorders.

#### 3.1.6. Bipolar Disorder (BP)

A cohort study of 267 young female adults with ASD were matched with 534 referents, and the hazard ratio for BP in ASD was 5.85 [61]. This gives a P(ASD|BP) of 0.272 or 27.2%. A study of 9062 children under 16 with ASD and 90,620 referents [68] gave an RR of 6, and this gives a P(ASD|BP) of 0.277 or 27.7%. The study did not give a gender breakdown but, in adolescents, BP has a M/F ratio of 1:1.3 [69], so this value is likely an underestimation.

#### 3.1.7. Schizophrenia Spectrum Disorder (SSD)

Published values for P(ASD|SSD) vary widely, likely due to significant diagnostic overshadowing, but the upper estimates are about 0.5 [70,71]. Overall P(SSD) is about 1% with a male/female ratio of 1.4:1 [71,72,73], giving a female P(SSD) of 0.0083. If we take P(SSD|ASD) to be 0.06 [74], then

P(ASD|SSD) = 0.434

This suggests that values for P(ASD|SSD) of up to 0.5 or 50% are plausible. This is supported by the shared genetics of SSD and ASD [75].

#### 3.1.8. Obsessive–Compulsive Disorder (OCD)

From a UK mental health trust study [76] of females aged 4–17, the number of females with OCD + ASD, OCD, and ASD are 121, 522, and 1625, respectively. Then, P(ASD|OCD) is 121/522, i.e., 0.2318 or 23.2%. This paper also gives a P(OCD|ASD) of 0.0745. With a female OCD prevalence of 0.015 [77], Bayes’ theorem gives a P(ASD) of 0.047, another independent measure of the high prevalence of female ASD. The relatively young age range may explain the value being at the low end of the overall calculated range of P(ASD).

#### 3.1.9. Anorexia Nervosa (AN)

There is reasonable current agreement [78,79] on a range of about 0.20 to 0.30 for P(ASD|AN). A value of 0.25 was used to calculate P(ASD) [18]. Margari et al. [80] give data to calculate a P(AN|ASD) of 0.068 compared to the published value [18] of 0.083. With the same values [18] for P(ASD|AN) of 0.25 and P(AN) of 0.02, this gives a P(ASD) of 0.074. This is the third-quartile value of the published median estimate of 0.060 and independently corroborates the estimated P(ASD). A further value of female P(AN|ASD) from Camm-Crosbie et al. [81] of 10.7% gives a P(ASD) of 0.047, at the lower end of the Bayesian range. The three values for P(AN|ASD) give a mean of 0.086 or 8.6%, much lower than the erroneous value of the inverse probability of 20–30% (Section 4.3).

#### 3.1.10. Borderline Personality Disorder (BPD)

The value is uncertain with many overlapping features and uncertainty about the age when the diagnosis should be considered, but 14.6% [82] is widely referenced, giving a P(ASD|BPD) of 0.146. This value was used to calculate a P(ASD) of 0.060 [6].

#### 3.1.11. Post-Traumatic Stress Disorder (PTSD)

PTSD is experienced by 10–12% of women [83]. There is not a precise figure for female P(PTSD|ASD), but a lifetime figure may be as high as 60% [84]. Then, we find

P(ASD|PTSD) = 0.30

Considering the stresses that autistic women have to cope with [23], it is not surprising that up to 30% of women with PTSD may have autism and will need to be diagnosed so the PTSD is not perpetuated by inappropriate therapy.

#### 3.1.12. Any Mental Health Disorder (MI)

Nyrenius et al. [85] give P(ASD|MI) as 18.9% in an adult outpatient psychiatric clinic. It was not gender-specific and the authors emphasized that the overall value including autistic traits could have been much higher. Since female rates are typically higher than male rates, this is definitely a low estimate. Studies of P(MI|ASD) are quite heterogeneous. LeCavalier et al. [86] give any externalizing disorder as 80.9% and any internalizing disorder as 43.6%. Lever et al. [87] give 79% with no gender breakdown, but again, female rates are typically higher than male rates. If we take 80% as indicative, then with the National Institute of Mental Health [88] 2021 value for the female P(MI) of 0.272, we obtain a P(ASD|MI) of 0.176 or 17.6%. These values of 18.9% and 17.6% are less than for some individual conditions because it is common with ASD to have multiple mental health comorbidities [89]. The outcome of the calculations of the proportion of women with comorbid ASD in mental illness are summarized in Table 2.

The overall magnitude of P(ASD|MI) reinforces the primary thesis that female ASD is being routinely missed in female mental illness. Nearly one in five women with mental illness appear to be autistic. Generally, P(MI|ASD), P(MI), and P(ASD) are secondary (2^0^) data and P(ASD|MI) can be calculated. P(ASD|MI) is occasionally available as a given value. If so, and P(MI) and P(MI|ASD) are also available as 2^0^ data, then another value of P(ASD) can be calculated. These additional results for P(ASD) were added to give overall values in Section 3.3.

### 3.2. Proportion of Female ASD in Conditions Consequent on or Associated with ASD and Female MI

#### 3.2.1. Attempted Suicide

While not a categorical mental illness, attempted suicide (AS) is an indicator of a serious underlying problem [17]. A Danish registry study by Kolves et al. [90] showed a marked increase in attempted suicides in female ASD, P(AS|ASD), compared to the non-ASD population, P(AS|not ASD). The comparison gave a likelihood ratio of 6.0. The LR was derived from AS per person–years, which gives a relative risk, so the resulting P(ASD|AS) of 0.277 would be the probability the patient on presentation has ASD. The clinician in practice has to first assess the patient by how they present, and then consider the variables that need to be diagnosed and managed. This probability of 27.7% strongly suggests that identifying ASD is essential in the management of female attempted suicide, with tailored prevention strategies [90]. If the presence of ASD is recognized in attempted suicide in treatment-resistant depression, the knowledge is, of itself, therapeutic [16].

#### 3.2.2. Completed Suicide

**Figure 5:**
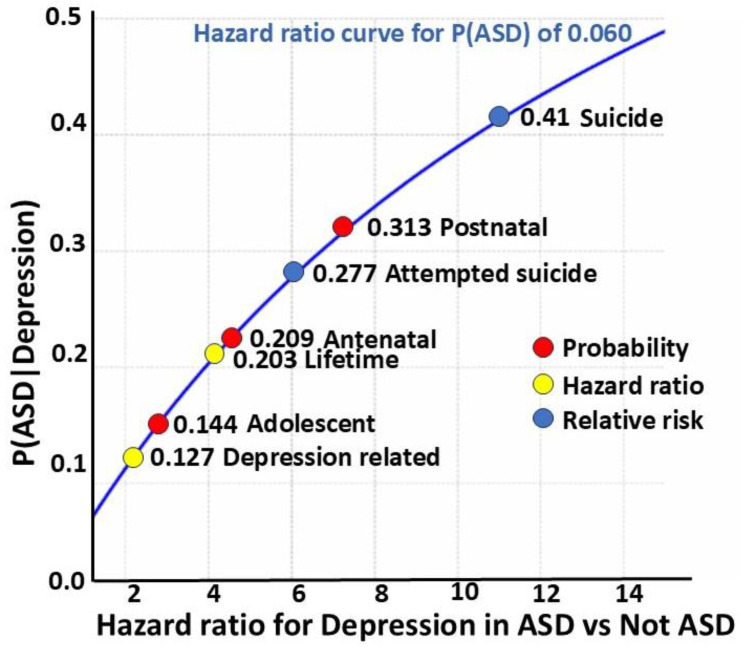
The relation of P(ASD|depression) to manifestations of depression.

The rate of completed suicide (CS) has been examined through the Swedish national register [91]. The rate in female ASD, P(CS|ASD), was much higher than in the general population, P(CS|not ASD), and even higher in high-functioning ASD. The relative risk for CS of the entire female ASD cohort compared to the control females was 0.0032/0.0003 or 10.667. This is P(CS|ASD)/P(CS|not ASD), mathematically equivalent to the LR and giving the P(ASD|CS) of 0.405, or 40.5%. A study of autism and autistic traits in those who died of suicide [92] gave a value for P(ASD|CS) of 0.414, very similar to the Bayesian calculation. The methodology, however, was completely different. The authors assessed coronial reports and medical records. There was no gender breakdown, but the risk of suicide in ASD may be higher in females than in males [91], and so a P(ASD|CS) value for females may be a low estimate. This is additional evidence that ASD increases the severity of comorbid mental illness. The close correlation of the values for suicide allows us to reverse engineer a value of P(ASD) of 0.062 in Appendix C. The relation of ASD with manifestations of depression is sobering (Figure 5).

The hazard ratio method is detailed in Appendix A. The proportion of autistic women increases as the clinical features of depression increase in severity and persistence. In this study, the proportion with postnatal depression was comparable to the proportion of attempted suicides. The American Psychiatric Association’s clinical review journal suggests ADHD as a risk factor for suicide but does not list ASD [93].

#### 3.2.3. Sexual Violence

Cazalis et al. [94] point out that being on the autistic spectrum is characterized by “experiencing difficulties in social communication such as decoding hidden intentions and emotions of others, understanding implicit communication and elements of context.” They found that 88.4% of autistic women had suffered sexual violence (SV), starting in two-thirds before the age of 18. This is compared to 27% of the general female population [95], giving a P(ASD|SV) of 0.196 or 19.6%. Consequences of sexual abuse before age 18 include depression, post-traumatic stress disorder, substance abuse, and suicide [96]. As for all the mental illnesses discussed, considering ASD on presentation would permit targeted interventions likely to be effective in neurodivergence. Childhood abuse is particularly important as an upstream root cause of the cascading downstream consequences.

#### 3.2.4. Sleep Problems

Gowin et al. [97] looked at sleep problems (SLPs) and the risk of suicidal behavior as preteens at age 10 transitioned to early adolescence at age 12. Increased suicidal behavior was associated with the severity of SLPs, insomnia and daytime somnolence, anxiety and depression, family conflict, and being female. Neurodivergence as a covariate was not considered. Estes et al. [98] examined SLPs in children aged 6 to 12 years. SLPs were present in 84.4% of girls with autism compared to 44.8% of typically developing girls. Anxiety was present in 41% of the autistic girls but the rate of SLPs was the same whether anxious or not, and so anxiety was of no value as a red flag for ASD. They concluded that SLPs in autistic females (SLP|ASD) should be carefully considered, and that an increased awareness of ASD in SLPs, i.e., P(ASD|SLP) could improve the identification of females on the autistic spectrum. Pediatric clinicians are very aware of P(SLP|ASD) since it is so common (0.844). An estimate of P(ASD|SLP) was not given. Then, P(ASD|SLP) = 0.113 or 11.3%.

While clinically significant due to the underestimation of P(ASD), it is still not very common in 6–12-year-old girls. During adolescence and into adult transition, it becomes much more problematic. Halstead et al. [99] found that 90% of autistic adults (sample 2/3 female) have clinical SLPs. A health professional had not been consulted in 64.4% of these cases, and of those who sought help, up to 80% had an unsatisfactory outcome. While the rate of SLPs in adult ASD is essentially unchanged from childhood, the rate for adult women overall has declined to 21.8% [100], and therefore P(ASD|SLP), by Bayes’ theorem, has increased to 24.8%, comparable to the categorical MI already listed in Table 2. Since the treatment of SLPs in women already diagnosed with ASD is unsatisfactory, it is inevitably going to be worse if the ASD has not been diagnosed. Unlike in the general population, the suicide mortality risk is higher for autistic females than males [101]. There is then a substantial population of females with ASD and SLPs (5.4% of women) with a high risk of suicide who are poorly recognized and ineffectively treated.

#### 3.2.5. Avoidant–Restrictive Food Intake Disorder

Avoidant–Restrictive Food Intake Disorder (ARFID) is a feeding and eating disorder recognized in DSM-5 that presents with substantial heterogeneity across the life span. There are three main drivers for the symptomatology:

1. Avoidance based on the sensory characteristics of food.
2. Apparent lack of interest in eating and in food.
3. Concern about adverse consequences of eating.

These features strongly suggest an overlap or comorbidity with ASD. However, a 2023 review of ARFID diagnostic assessments [102] did not consider ASD as a factor. A recent and likely reliable population estimate of female ARFID prevalence P(ARF) gave a value of 0.0249 or 2.49% [103]. P(ARF|ASD) in children of all genders has been recently assessed by Koomar et al. [104] to be 0.21 or 21%. The ARFID MFOR is likely to be 1:1.7 [102], which suggests that a Bayesian calculation of P(ASD|ARF) will underestimate the proportion in females. Then, a lower limit for P(ASD|ARF) will be

P(ASD|ARF) = 0.21 × 0.060/0.0249 = 0.506 or 50.6%

We can estimate female P(ARF|ASD) in the Koomar study. The Simons Foundation Powering Autism Research for Knowledge (SPARK) data providing the overall 0.21 value gave an ASD MFOR of 1.71:1 [56]. Let the male value be *m*, then, to deconstruct the P(ARF) weighted average as follows:

(1.71 × *m* + 1 × 1.7*m*)/(1.7 + 1) = 0.21

*m* = 0.1669

If we assume that the ARFID MFOR is the same for ASD females as for the general female population, then

Female P(ARF|ASD) = 1.7 × 0.1669 = 0.2837

By Bayes’ theorem,

Female P(ASD|ARF) = 0.2837 × 0.060/0.0249 = 0.6834 or 68.3%

Haidar et al. [105] reported 139 cases of ASD in 319 cases of ARFID from the British Pediatric Surveillance Unit and the Child and Adolescent Psychiatry Surveillance System in 2024. ARFID males represented 54.5% and females 45.5%. The median age was 11.9 years, in the range of 5–18. The percentage with ASD was then 43.57%. No gender ratio for the ASD cases was provided, but we can estimate the female P(ASD|ARF) using the ARFID MFOR [103], expressed as 0.558:1. Then, let female P(ASD|ARF) be *f*. Then

0.588*f* × 0.545 + *f* × 0.455 = 0.4357

0.7755*f* = 0.4357

P(ASD|ARF) = 0.5618 or 56.2%

In 2025, Wronski et al. [106] reviewed the relation between ARFID and mental and somatic conditions from the Swedish National Patient Register from 2004 to 2020. They found a Cox regression hazard ratio for ASD of 12.10, giving a P(ASD|ARF) of 0.436. In Appendix A, we find that the hazard ratio underestimates the value by ~15%, giving a likely P(ASD|ARF) of ~0.501 or 50%. These values lie within the first Bayesian calculated range. As expected clinically, a very high proportion of girls with ARFID, at least 40%, and probably higher, appear to be autistic, and it is essential to consider the diagnosis to provide optimum intervention.

### 3.3. Validation of the Female Prevalence Value for P(ASD)

**Figure 6:**
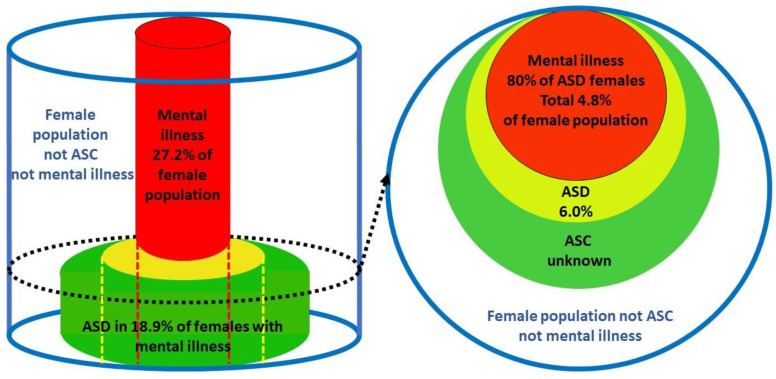
Three-dimensional Euler diagram and cross section of female population prevalences.

The relation between autism and mental illness in the female population is summarized in Figure 6.

We can use these data to obtain another independent value for P(ASD) using Bayes’ theorem:

P(ASD) = P(ASD|MI) × P(MI)/P(MI|ASD)

=0.189 × 0.272/0.80

=0.064

In addition to the 14 values already determined [18], we can now add 0.064, from Section 3.1.8, 0.047, from Section 3.1.9, 0.047 and 0.074, and from Section 3.2.2, 0.062. The median value of P(ASD) is then 0.060, with Q1 0.055, Q3 0.074, IQR 0.019, range 0.047–0.094, and number of values 19.

For individual mental illnesses, values for P(MI|ASD) and P(MI) are quite variable due to heterogeneous populations, different diagnostic methods, and diagnostic overshadowing. Average values for an individual comorbid illness P(MI|ASD) and mental illness P(MI) are going to be very different due to the natural variation in the prevalence of the different mental illnesses. The degree to which they are comorbid with ASD is going to vary, and there are different degrees and combinations of multiple comorbidities in individual patients. All these variations are integrated in the variables for overall female mental illness, i.e., P(MI|ASD), P(ASD|MI), and P(MI), shown in Figure 6. The value of P(ASD) derived from the data for overall MI was 0.064. The mean for the other 18 measurements of P(ASD) was 0.063.

The two general methods [6,18] of determining P(ASD), by bias calculation from the author’s database and Bayes’ theorem from secondary peer-reviewed data, employ completely independent mathematical processes. The technique of synthesis is ensemble averaging [6], used to improve the signal-to-noise ratio by combining repeated methods of observing a single dataset (the bias results) with a single method of observing multiple datasets (the Bayesian results). A comparison of the results is shown in Table 3.

**Figure 7:**
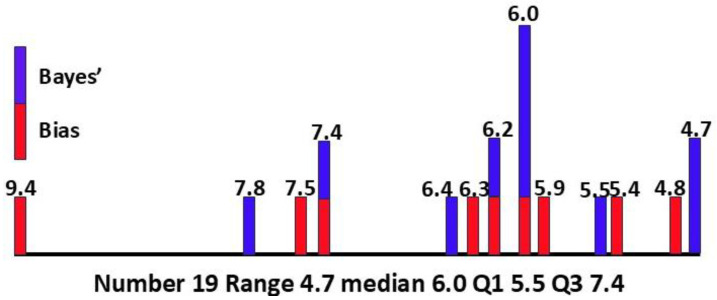
Calculated values of P(ASD) as percentages.

The overall calculated values and descriptive statistics for P(ASD) are shown in Figure 7.

The obvious strong correlation means that the Bayesian methodology alone is adequate to refine the P(ASD) value as more secondary data become available. The Bayesian methodology depends on data for females only, without comparison with male data. It relies on fewer assumptions than the bias method. The Bayesian values have quite a narrow interquartile range of 0.009. The results suggest that the overall median of 6.0% and mean of 6.3% for the prevalence of female ASD are plausible. The combined values are skewed to the high end of the range, so the median was preferred for calculating P(ASD|MI), giving more conservative values.

ASD is a complex outcome of multiple genes and environmental factors including epigenetics. This will produce a continuous transition between the neurodivergent and the neurotypical. It does appear that it is a clinical problem for about 6% of women.

This finding, again, should generate a number of possible research studies. We will now discuss the cycle that must be broken.

## 4. Discussion

### 4.1. Intervening in the Intergenerational Cycle

#### 4.1.1. The Cycle

What do we mean by a cycle? If we are considering a cycle of mental illness exacerbated by comorbid ASD, the cycle is the intergenerational problem of mental illness, but the generations are obviously linear. You break the cycle by intervening in a linear process. The mother is central in that she participates in nature and nurture. The father does as well, but nurture early in development, in particular, centers on the mother in human society, and this paper has explored the relation between female autism and mental illness. Intervention, in principle, could equally apply to males, but the special case of PND in the mother will be highlighted. The problem considering males is that diagnostic overshadowing of male autism has been less explored, and an unbiased prevalence of male autism, required by the methods used in this paper, has not been obtained. A preliminary attempt at a solution is, however, provided in Section 6.

#### 4.1.2. Regulation of Development

Brain development is choreographed by complex gene programs, regulated in turn by epigenetic mechanisms. The epigenome sits at the interface between brain development and the environment, allowing the epigenome to fine-tune gene expression in response to physiological needs. Early life stresses alter genes that regulate epigenetic function itself, heightening vulnerability to neuropsychiatric disorders. The epigenome continues to mature postnatally, consisting of cell type-specific patterns of DNA methylation, chromatin modifications, and non-coding RNAs. It is largely complete by the periadolescent period. Epigenetic mechanisms help govern both critical-period and lifelong plasticity [107]. In puberty, the hormonal surge acts as a catalyst for neural remodeling, with synaptic pruning, myelination, and the restructuring of neural networks. Prolonged plasticity of the prefrontal cortex allows adaptation to changing contexts but also becomes more vulnerable to environmental stressors and resulting anxiety and depression [108].

#### 4.1.3. The Scenario

**Figure 8:**
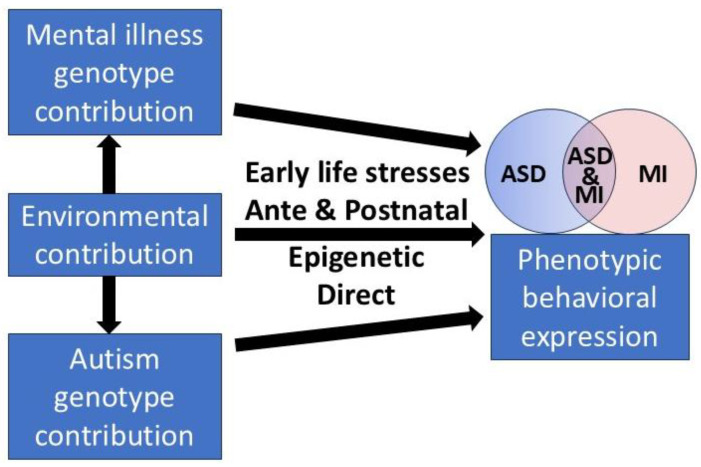
Schematic relation between genes and the environment for ASD and MI.

Any overshadowing mental illness could be chosen, but the most urgent, which also has a clear path to management, is postnatal depression. Let us enter the cycle with a pregnant young autistic woman. Her autism is undiagnosed, as is her antenatal depression. She develops visible PND but her autism remains hidden. This causes prolonged PND, resistant to treatment, and exposes her child to developmental risks, which vary with the persistence of stressful exposure from early sensorimotor white matter tract impacts to later interregional connectivity problems. Both relate to PTSD [109]. The mother’s depression may last for several years [110], and the effects include behavior problems, lower math skills, and adolescent depression [111]. Epigenetic effects of stress in early life include effects on genes for the glucocorticoid receptor, brain-derived neurotropic factor, and serotonin transporter and may lead to later major depression, generalized anxiety, and schizophrenia [112]. There is no published measurement of the risk of autism in the child of an autistic mother, though it was quite common to see this in my clinic. Independent of autism in the mother, if a child has autism, the risk in a sibling is about 20% [113]. An autistic child of a depressed mother faces the double jeopardy of the effects of maternal depression and his or her autism. If the mother has autism as well, her depression is likely to be more severe and prolonged, again compounding the problems for the child. Since most mental illnesses start in young people (Section 3.1.1 and Figure 4), the detection of autism at any generational level will help normalize child development. For the daughter with autism, bullying at school is a particular problem. The bullying of any child causes an increased risk of depression, anxiety, and suicide, and school avoidance, truancy, and dropping out of school will exacerbate these mental health issues [114]. Autistic children are victimized more, and girls, being more socially motivated than boys, are at higher risk [115]. For all these reasons, the red flag of PND is the opportunity to diagnose autism in the mother and child, and to manage and reduce the cascading consequences for both physical and mental health over the generations. The generalized relations are demonstrated in Figure 8.

*Direct* refers to the environmental contributors to autism with toxic effects on neurodevelopment other than via epigenetics, such as sodium valproate during pregnancy [116]. They are largely unknown, but of note are some infectious agents during pregnancy that can cause autism in the child. Of particular interest is congenital rubella [117]. It is ironic that with the antivaccination hysteria induced by the fraudulent MMR study, MMR immunization is protective against one cause of autism. Bayes’ theorem can also determine the proportion of autism from exposure to particular agents. The risk for acetaminophen (paracetamol) is examined in Appendix D.

### 4.2. Stumbling Blocks

In adults, identification is not made and services are not accessed [118]. Neurotypical people tend to have less-positive first impressions of autistic people [119], most commonly because they appear awkward and lacking in empathy. Health workers are not necessarily immune [11]. ASD should be considered a possible underlying condition in adult psychiatry. There is no easy way of ruling out ASD in this population [120,121]. For anxiety and depression, more session time is needed. More medication is used in the ASD comorbid group, implying unresponsiveness to talk therapy or more complexity [122].

Malik-Soni et al. [37] find that the transition to adult ASD services has multiple problems, as follows:

1. Services are accessed by only 1/5 of youths with autism.
2. About 70% of pediatricians do not support youths during the transition process and >50% of families lack information on how to proceed.
3. Little is still known about the effects of comorbid MI. Adult physicians need to monitor ongoing symptoms of ASD—which can intensify and diminish—to guide diagnosis and treatment choices.
4. Specific barriers include a shortage of health care services, poor physician knowledge, cost of services, lack of family and individual knowledge, stigma, and language barriers.

We would add that language here refers to non-native speaking patients and does not include double empathy problems with a lack of mutual understanding in one’s native language.

### 4.3. Conditional Probability: Transposing the Conditional

When searching for the probability of ASD in a particular mental illness, it was routinely found that the value provided was the probability of the mental illness in ASD. Generally, as this paper has shown, there were no data available, and the search engine should have responded that there was no information. Thus, the response was wrong, or at best ambiguous. This mistake, transposing the conditional, is unfortunately very common. The transposition can potentially have serious consequences. For example, the proportion of female patients with anorexia nervosa believed to be autistic (P(ASD|AN) is well-established to be about 20–30% [78,79], but this information is often given under a heading of the likelihood of developing AN if you are autistic P(AN|ASD). This could lead an autistic teenage girl to incorrectly believe she has a high chance of developing AN, the MI with the highest mortality [123] and only a 20% chance of complete recovery [124]. With a high level of anxiety, depression, and food sensitivity to deal with already, this would not be helpful. If we take P(AN) as 0.02 and P(ASD|AN) as 0.25 [18], then P(AN|ASD) is 0.083 or 8.3%. There is corroboration in the literature of 0.068, or 6.8% [80] and 10.7% [81] for an overall mean of 8.6% (Section 3.1.9). While higher than in the general female population, it is still substantially lower than the value she would be led to believe. We can also surmise that the future is much more hopeful with the new information on the different causes of the comorbid AN [11,36,50] and the improved outcome with therapy which recognizes neurodiversity [125]. Ironically, the problem here is the unrecognized P(AN|ASD), the reverse of the usually unknown P(ASD|MI). The understanding of the logic of conditional probability remains poor. It needs to be continually reinforced [126].

### 4.4. Effective Therapy

The key to effective therapy is understanding neurodiversity. The categories of neurodivergent and neurotypical just represent different ways of thinking. Neurodivergence is not necessarily inferior and may only be disabling due to mutual misunderstanding in an overwhelmingly neurotypical environment. Understanding neurodivergence is critical for helping autistic adults cope in all walks of life, including health [127]. The World Health Organization’s definition of health requires “a state of complete physical, mental and social wellbeing…” [128]. ASD does not meet this definition of health, but the definition should be accepted for ASC (Section 1.5). The neurodivergent minority is going to struggle in a predominantly neurotypical world, so assistance must be twofold; to optimize social adaptation to that environment, but also to treat comorbid mental illnesses in a manner empathetic to neurodivergence. A healthy outcome does not, however, require conversion to a neurotypical mental state. Neurodiversity recognizes that disability needs attention but also recognizes that, in particular areas, neurodivergence can have great strengths [129] which can be empowering and thereby, if necessary, therapeutic. Neurodiversity-affirming interventions are essential [130]. A recent Delphi study of the management of comorbid ASD and AN emphasized the need to manage the individual in the context of ASD and that treatment goals should not aim to change autistic behaviors [131]. The understanding of the importance of the empathetic management of comorbid ASD in MI remains embryonic. A 2024 study [132] of outpatient MI presentations in 13–24-year-olds excluded ASD from consideration.

Rules for empathetic therapy include the following. Recognize the centrality of the double empathy problem [133]. There are variations in empathy in autistic people, and some individuals can be quite empathetic [134]. Recognize that autistic people are often more deliberative and less intuitive than neurotypical individuals [135]. Cognitive behavior therapy, for example, can be adapted to the core features of ASD [136]. In designing therapy improvements, researchers with lived experience of autism should be involved [137]. We would add the inclusion of autistic clinicians, patients, and parents.

Specific areas for therapists suggested by Gilmore et al. [138] include the following:

1. Be a change agent in the mental health workplace.
2. Make thoughtful language choices.
3. Individualize treatment.
4. Leverage patient strengths.
5. Agree on practical goals in navigating life situations.

We would add that a necessary corollary to individualizing treatment is individual treatment. Autistic people are often uncomfortable in a group, and trying to stretch therapists’ resources is pointless if the therapy will not work.

### 4.5. The Argument for Implementing a Screening Program

The results presented in this study suggest that the qualitative assessment of risk (frequency [139] × severity [7,12,13]) is high. The problem in the past was that ASD overshadowed in mental illness had not been considered to be quantitatively significant, especially in adolescent and young female adults. Papers which report values for P(MI|ASD) call for searching for ASD in MI without being able to give a value for P(ASD|MI). This concern is predicated on an assumed prevalence of female ASD of about 1%. With a value of 6%, it would be anticipated that all interested clinicians would recommend at least a screen for all women with a mental illness, and a more in-depth assessment where the treatment progress was not satisfactory. With the dearth of secondary data to give a more complete picture, the prudent course for the clinician would be to consider ASD in any individual with a mental health issue. Different jurisdictions will deal with the problem by different diagnostic methods and treatment options, but the common key to success is recognizing the necessity of managing neurodivergence in a specifically empathetic manner.

WHO principles of a successful screening program [140] are listed against ASD/MI comorbidity:

The targeted population.

A targeted screen of women with mental illness. The type and severity would depend on jurisdictional judgment. Perinatal depression would be an obvious choice.

Condition.

1. *The condition should be an important health problem*.
  This is common with significant intergenerational morbidity.
2. *There should be a recognizable latent or early symptomatic stage*.
  There are multiple routes to early diagnosis once the need to search is recognized.
3. The natural history of the condition, including development from latent to declared disease, should be adequately understood.

The natural history is well understood.

Test.

1. *There should be a suitable test or examination*.
  There are multiple diagnostic pathways.
2. *The test should be acceptable to the population*.

The specific population often has an intractable condition and would generally welcome assessment [141].

Treatment.

1. *There should be an accepted treatment for patients with recognized disease*.

There are multiple therapeutic options once ASD is recognized.

Screening Program.

1. *There should be an agreed policy on whom to treat as patients*.

Whom to assess may vary by jurisdiction, but all those diagnosed should be offered treatment. The knowledge of the correct diagnostic formulation may be enough therapy for some patients.

2. *Facilities for diagnosis and treatment should be available*.

Facilities are generally available but often under-resourced in funding and trained practitioners.

3. *The cost of case-findings (including the diagnosis and treatment of patients diagnosed) should be economically balanced in relation to possible expenditure on medical care as a whole*.

The preliminary Pareto analysis demonstrates that management is likely to be very cost-effective.

4. *Case-findings should be a continuing process and not a ‘once and for all’ project*.

This will depend on the jurisdiction and outcome of pilot programs.

### 4.6. The Art of the Possible

We must trust that responsible governments will adopt a utilitarian approach and generally provide services doing the most good for the greatest number of people. To do this, they require an evidence base, in particular if the service has received little attention in the past. It is not strictly true that management always requires measurement [142], but if you want evidence-based policy, it helps to provide quantitative evidence, otherwise governments have a tendency to find policy-based evidence [143], and policy based on evidence and evidence based on policy are definitely not commutative. If governments have evidence, they tend to apply risk management. ASD is common in young women with mental health issues, a population at risk. The commonly occurring ASD increases the severity of their condition, and so the risk, the product of frequency and severity of outcome, is high. Since female ASD is cryptic, the obvious next step is a screening process, as outlined above. There are effective screening mechanisms which can be incorporated in normal care [48] once the possibility that ASD may be present has been recognized [6]. There is a clear road map for effective therapy (Section 4.4), and it is cost-effective (Appendix B.3).

The neglect of female ASD in adult mental health has been due to inertia in knowledge transfer. There is no obvious political issue, and the solution lies within the health field itself. As the Pareto examination indicates, it is possible to achieve a significant improvement in mental health coverage without extra costs. If the knowledge is transferred, the stumbling block will be the training of extra practitioners and getting them to travel to the regions, but you have to start somewhere, and the following section shows how some difficulties may be overcome.

### 4.7. It Takes a Village

According to the African tradition, it takes a village to provide a safe, healthy environment for children [144]. This conveys the message that it takes many cooperating people to provide complete childcare. Two elements relevant to health are social connectedness, where different agencies can cooperate, and the breaking down of siloes within the complex agencies of health delivery. This is much easier to achieve in…a village. Three examples follow from my own personal experience of rural pediatric practice. During a period of serving as the sole pediatrician in a rural population of about 50,000, about a third of my patients were seen for behavioral problems alone and a further quarter had physical illnesses complicated by behavioral problems. It was essential and not too difficult to get to know teachers, guidance officers, principals, and education bureaucrats operating in the local provincial area. This was crucial for the effective management of ASD, which was also my task to diagnose. It was easier to have a personal relationship with the local child-protection service and have influence in their decisions as the only pediatrician on their team. Health is notorious for siloes, but a common interest in preventing birthing disasters and ensuring optimal outcomes for mothers and babies led to a close working relationship with the local midwives. Considering the new knowledge regarding perinatal depression (Section 3.1.3), if midwives were screening for maternal ASD [145], a logical step forward would be to use local pediatric expertise to at least informally assess mothers. This would, in turn, improve the downstream pediatric care of the children. There is no reason why this could not extend to antenatal care. This would assist mothers during delivery with an autism-friendly approach [48,145]. Even an informal diagnosis can be very therapeutic and would likely decrease the degree of depression. There is still likely to be a problem with allied health access, i.e., psychology, speech therapy, and occupational therapy, but there is evidence that telehealth is not inferior to face-to-face therapy [146]. Counterintuitively, non-acute care delivered in a rural setting can often be superior to that in larger centers due to better interdisciplinary communication, including across the pediatric/adult divide. In larger centers, from my experience as a clinician and medical executive participating in hospital design, the main problem is the spatial isolation of busy clinicians. In university teaching hospitals, a campus including adult, children’s, and women’s hospitals maximizes interdisciplinary communication. Mental health facilities should also be on campus. Clinics and consulting rooms on site will mean that clinical staff can participate in seminars, and be on site if there is an emergency with one of their patients. Staff lounges and dining facilities may be seen as elitist but are a very efficient way to share confidential information. They should be interdisciplinary but reserved for clinicians. The main seminar room needs to be central so that staff can get there and back during their lunch break. For smaller district facilities, the same design rules apply, appropriate for scale. The common and pervasive root cause in any setting is inadequate interdisciplinary communication. It must be made as convenient as possible.

### 4.8. The Long View

There are too many stresses facing young people today [147,148]. World-wide or jurisdiction-wide stresses are beyond easy control, but managing the interacting effects of the common problem of comorbid ASD and stress-related MI is quite feasible at the health service level with current techniques. Solving what is solvable boosts morale by showing that we care. From Chapter 63 of the “Tao Te Ching” of Lao Tzu [149],

“*Difficult problems are best solved when they are easy.*

*Great projects are best started when they are small.*

*The Master never takes on more than she can handle,*

*Which means she leaves nothing undone.*”

### 4.9. Overview

This study has used novel methods to organize existing data in order to open a new window for clinical action in a difficult area of mental health affecting young people and provides a new frame of reference for the epidemiology of autistic females. It has, for the first time, quantified the proportion of women with several mental health problems who are autistic. The structure of the paper develops a coherent system of rules to solve similar problems with current disparate incomplete data sources and demonstrates what necessary data are missing. It establishes the extent of the problem, what modifications are needed in established therapies, and suggests new solutions, in particular in cooperative management between different health professionals. Financial resources and mental health therapist numbers are quite inadequate, and appropriate attention will free up therapy time to deal with more patients and devote more time to reducing their intractable problems. A recurring theme among patients and their parents is that health professionals did not listen to them. The mother of one of my patients said: “*We have to learn to think aspie*.” This is a beautifully succinct statement of the double empathy problem. We all, especially if neurotypical, have to get better at listening, empathizing with different mindsets, and managing the problems while celebrating the benefits of neurodiversity [150].

## 5. Limitations of the Study

The major limitations are as follows:

The author is the only researcher who appears to have used these methods. While Bayes’ theorem is well established, the HR- and RR-as-LR version for this purpose of quantifying an overshadowed condition and the Pareto equations are novel. There is a need for the external validation of the results in prospectively recruited clinical samples. The P(ASD|MI) estimates rely on secondary sources with heterogeneous case ascertainment, and camouflaging/diagnostic overshadowing may bias co-occurrence downwards, as explained in Appendix A. Estimations may vary across health systems depending on the diagnostic criteria used. The recommendations of researchers who have estimated P(MI|ASD) with a presumed P(ASD) of ~1% suggest that there is no lower bound to assessing P(ASD) in MI, but there may well be resource constraints in different jurisdictions. The Pareto assessment does, however, suggest that making management more efficient by making the diagnosis should save on resources.

The experience of adult women in struggling to obtain a diagnosis of ASD and the evidence for extensive female camouflaging makes it certain that the prevalence must be significantly higher than generally reported. When Bayes’ theorem is used with reliable estimates of mental illness prevalence in diagnosed ASD populations, and when ASD has been explicitly sought in diagnosed MI populations, the value of P(ASD) should be accurate with the probability equation, which is mathematically exact. The hazard ratio method will vary over time with diagnosis rates, and gives a conservative value, but has the advantage that biases in diagnosis have a limited effect on the result (Appendix A). Secondary data is patchy, but it turns out that there is quite a lot of agreement for a prevalence of ~6% (Section 3.3, Table 3). In particular, the two Bayesian methods give quite a narrow band of results. However, this needs to be independently confirmed.

Estimates of P(MI|ASD) are heterogeneous with diagnostic overshadowing. Estimates of P(MI) are also heterogeneous with differing values due to diagnostic variation and the prevalence being different at different ages. As a result, values for P(ASD|MI) are indicative at this stage. However, as seen in Table 2, results by different methods, and with different secondary data values by different authors, do tend to correlate.

The Pareto analysis is novel. With an improvement in the diagnostic rate of ASD and consequently more efficient management of the comorbid conditions, there should be savings in overall patient populations, but this analysis is a first attempt at a method of quantification. Current estimates are novel and would be refined as more extensive secondary data become available. The strategy of the study is to shift the frame of reference of ASD comorbidity, and it is very early days for costings.

## 6. Future Directions

Due to the underestimation of P(ASD) and the very recent appreciation of the deleterious effect of ASD on comorbid MI, the level of risk has been seriously underestimated. It is therefore not surprising that knowledge of the degree of risk has not reached our adult colleagues. With a new appreciation of the clinical problem, advances in many areas can be anticipated.

The value for P(ASD) looks fairly reliable. The qualitative reports on camouflaging suggest that we are missing a considerable proportion of females. It is reassuring that the use of Bayes’ theorem with variables from a number of independent secondary data sources do give a fairly narrow range of results that far exceeds the generally quoted estimate of 1%. Two different methods of calculation agree, but the value needs further corroboration by independent sources.

A refinement of diagnostic criteria will help quantify prevalence estimates. It is possible that the problem of diagnostic overshadowing may be resolved by adopting a continuous model of mental health conditions, as genetics suggests. This should simplify the management of comorbidity if that model is seen as the norm. Treatments could be applied to the entire continuum, as is becoming more common for ASD/ADHD in its new identity as AuDHD.

Data allowing for the calculation of the P(ASD|MI) for specific mental illnesses are sparse. As more values for P(MI|ASD) and P(MI), or P(MI|ASD)/P(MI|not ASD) become available, they can be assessed by Bayes’ theorem. Future values are unlikely to be low enough to obviate the need for routine assessment for ASD.

Recent rapid advances in knowledge about female autism should help break down the transition barrier from adolescents to adults.

More work is needed to continue to improve talking therapies in mental illness with comorbid ASD.

More examples of savings by managing comorbid ASD are needed. The Pareto method can be refined to quantify efficiency gains. Funding should follow quantitation of the risk of comorbid ASD in female mental health if management is clearly shown to work. Government tends to fund programs with evidence of success.

Can the methodology be used to find the true prevalence of ASD in boys with a mental illness? The Bayesian methods are applicable for any condition where there is diagnostic overshadowing by another condition. The first requirement is for an accurate population prevalence of the possibly overshadowed condition, here P(ASD) for males. We must find sufficiently reliable data for the prevalence of an overshadowing condition in the overshadowed condition and its inverse. Oppositional defiant disorder (ODD) can provide an estimate. We require P(ODD|ASD), the inverse conditional probability P(ASD|ODD), and the prevalence of the overshadowing condition P(ODD). There is the same dearth of gendered data as for females. However, a preliminary estimate is possible. The case of male ASD prevalence is interesting. Obviously, it is easier to diagnose, with a (biased) MFOR now of about 3:1. This does not rule out biases in males leading to overshadowing. My clinical experience suggests that there is still an underestimation of male ASD. The knowledge that AuDHD is common is still fairly new. The diagnosis of ADHD with comorbid ODD is a common clinical endpoint diagnosis for boys. It has unfortunate results, particularly at school, with a typical sequence of detention, suspension, and expulsion. The referral of these boys to my clinic when treatment was unsuccessful was quite common, and comorbid ASD was routinely sought. DSM-5-TR criteria for ASD were nearly always present. In 2015, Mayes et al. [151] looked for the presence of ASD in children with disruptive mood dysregulation disorder (DMDD), a new entrant in DSM-5 in 2013. They found, inter alia, that 91% of children with DMDD met the criteria for ODD. There were 582 children with autism in their sample, and 82.6% were male. Nearly all the children with autism had features of ADHD and 51% of the children with autism had ODD. No gender data was given. The age range was 6–16, and while in younger children ODD is more common in boys than in girls, in the teenage years the girls tend to catch up, according to DSM-5-TR. Hence, we will assume a low estimate for the percentage for boys at 51%, since the sample was also largely boys (82.6%). The values given for ODD prevalence vary widely but the prevalence given in DSM-5-TR is 3.3%. Their threshold criterion for diagnosis requires distress or negative impact rather than the clinically significant impairment required for ASD, but it ensures the behavior pattern is maladaptive or disruptive beyond normal developmental variation, so is clinically equivalent. Since my specialist clinic was assessing children referred by their general practitioner (family physician), the value of 3.3% is likely appropriate. My estimate for the proportion of boys with ADHD/ODD assessed as having ASD was about 90%. We then have a P(ODD) of 0.033, a P(ASD|ODD) of 0.9, and a P(ODD|ASD) of 0.51. By Bayes’ theorem, this gives a P(ASD) for boys of 0.058 or 5.8%. This value is very close to the median estimate of 6.0% for girls, as might be expected for a condition that is largely genetic [6,7]. Note that values for ADHD and the current estimate of P(ASD) for boys of about 4% are not needed for the calculation. It does mean that about 1/3 of boys may be misdiagnosed due to diagnostic overshadowing. This would have unfortunate consequences if true, because neurodivergent people need neurodivergent therapy. Another conclusion is that ODD might be nearly always a behavioral manifestation of ASD rather than a stand-alone condition.

## 7. Conclusions

This study completes a sequence of the following:

Quantifying the strong clinical suspicion of bias in detecting female ASD [6].
Finding the true value of female ASD prevalence implicit in that suspicion [18].

Calculating the practical clinical outcomes of a new frame of reference for the relation between ASD and female mental illness and a framework for data collection. This has been the contribution of this paper, with the following conclusions:

A median P(ASD) of 6.0% has received further confirmation.
Female camouflaging appears to begin at a very early age.

This prevalence of ASD in female mental illness is clinically significant at a population level and for individual diagnosis. It does seem likely that up to one in five women with a mental illness is autistic, and this is not generally being managed.

ASD in this population must be diligently screened for both prior to and after the transition to adult care.

The solution to facilitating this transition lies within the health system, in terms of establishing the extent of the problem and transmitting the information. The overall risk in the extent and severity of outcome of the ASD/MI comorbidities is much higher than currently understood.

The risk for comorbid depression and its consequences is of particular concern and should be urgently addressed. Intergenerational morbidity arises from both hereditary and environmental factors, and a timely diagnosis of girls and young women would be of high societal benefit.

Effective therapy is quite feasible. At the present time, a lot of energy is being wasted on ineffective therapy due to the lack of an ASD diagnosis. That wasted energy can be redirected to effective modes of management with a consequent large positive gain in efficiency.

The method of finding high-risk ASD patients has identified and quantified the drivers of the Pareto analysis, and the Pareto formulation has given an estimate of system gain and a framework for data collection.

The overall improvement in female mental health and prevention of the downstream and intergenerational effects of mental illness is likely to be substantial, but an accurate estimation of system gains is, at present, in its infancy.

The key to effective diagnosis and therapy is listening, understanding, and empathizing with neurodiverse individuals.

## Figures and Tables

**Table 1:** Onset of camouflaging behavior in girls.

Age of onset in years	3	4	5	6	7	8	9	10	11	12	13	14	15	16	17
Number = 82	1	4	8	16	9	7	5	13	5	4	3	1	2	2	2

**Table 2:** Summary of P(ASD|MI) values.

Condition	P(ASD|MI)	2^0^ References	Given Value or Bayes’ Calculation
Depression	0.127 0.144 0.203	18, 61 6, 62 18, 63	Bayes’ odds Bayes’ probability Bayes’ odds
Postnatal Depression	0.313	6, 64, 65	Bayes’ probability
Anxiety Disorders Social Anxiety Disorder	0.157 0.170 0.262	18, 61 18, 66 6, 67	Bayes’ odds Bayes’ odds Bayes’ probability
Bipolar Disorder	0.272 0.277	18, 61 18, 68	Bayes’ odds Bayes’ odds
Schizophrenia Spectrum D	0.434	6, 70–75	Bayes’ probability
Obsessive–Compulsive D	0.232	6, 76, 77	Given
Anorexia Nervosa	0.20–0.30 0.23	78 79	Given Given
Borderline Personality D	0.146	81	Given
Post-Traumatic Stress D	0.30	6, 82, 83	Bayes’ probability
Any Mental Illness	0.189	84	Given
	0.176	6, 85–87	Bayes’ probability

**Table 3:** Median and mean values of P(ASD) calculations.

Method	Number	Range	Median	1st Quartile	3rd Quartile	Mean
Bias	9	0.048–0.094	0.062	0.0565	0.074	0.065
Bayes’	10	0.047–0.078	0.060	0.055	0.064	0.061
Ensemble	19	0.047–0.094	0.060	0.055	0.074	0.063

**Figure A1:**
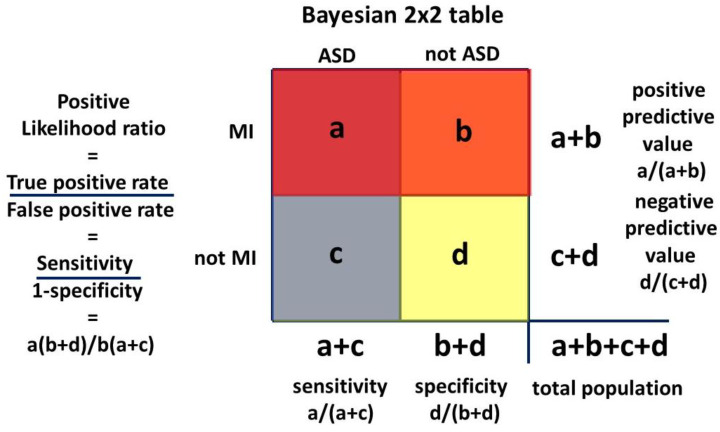
Venn diagram transformed into a 2 × 2 table. Quadrants: (a) MI and ASD, (b) MI and not ASD, (c) ASD and not MI, and (d) not ASD and not MI.

**Figure A2:**
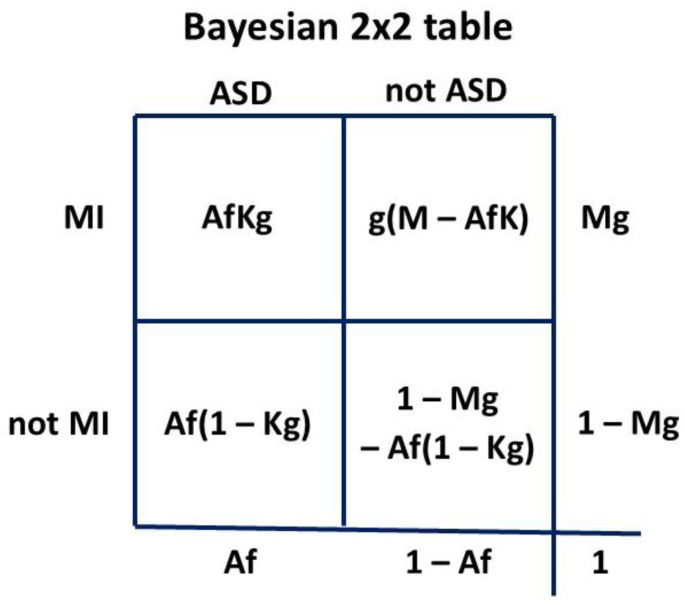
2 × 2 table to calculate the general LR formula.

**Figure A3:**
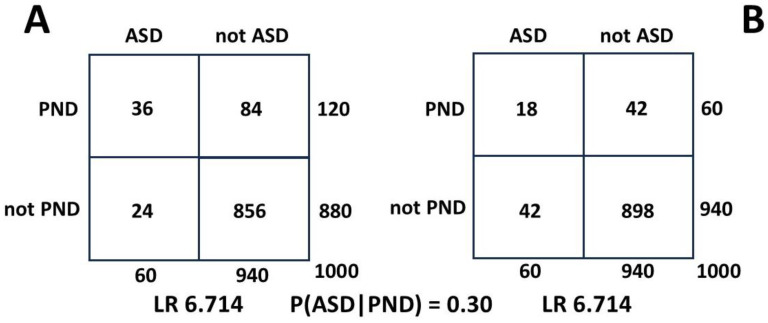
The effect of halving the measured MI on LR.

**Figure A4:**
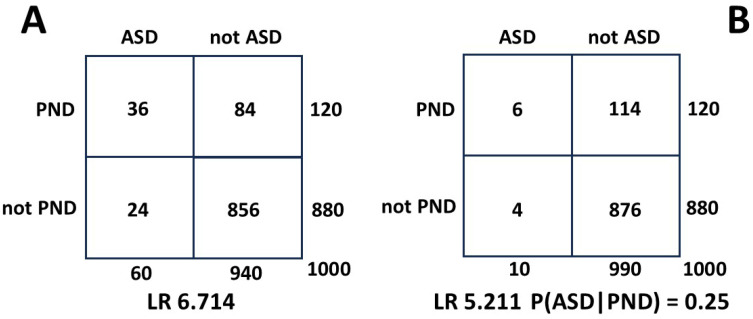
The effect of reducing the measured ASD by six times on LR.

**Figure A5:**
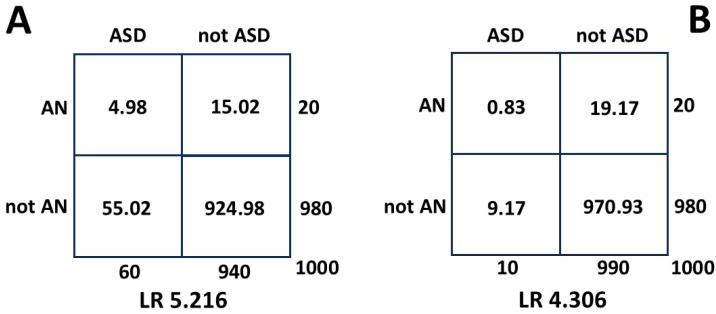
The effect of reducing both the true MI and measured ASD by six times on LR.

**Figure A6:**
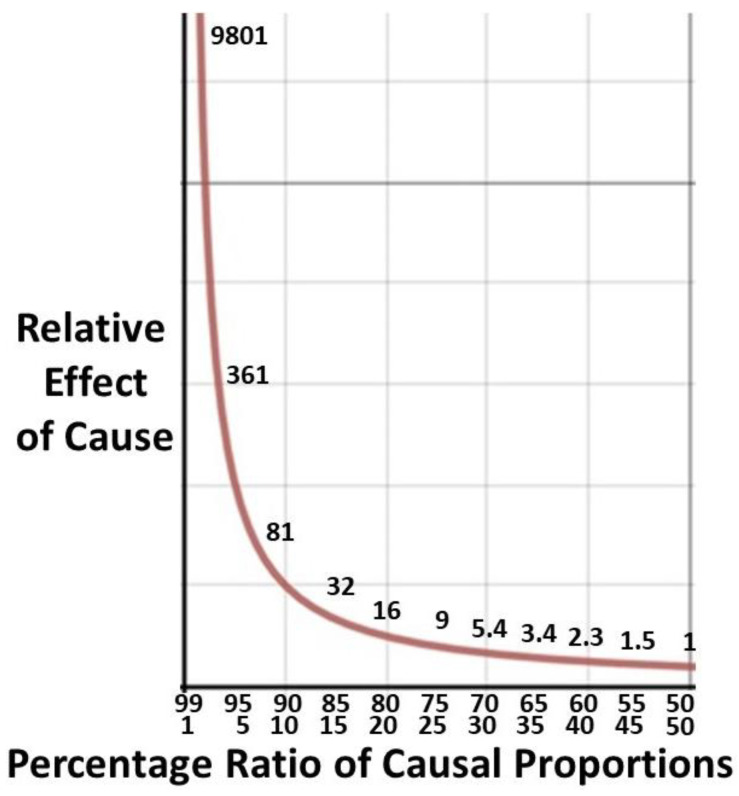
The curve of cause and effect.

**Figure A7:**
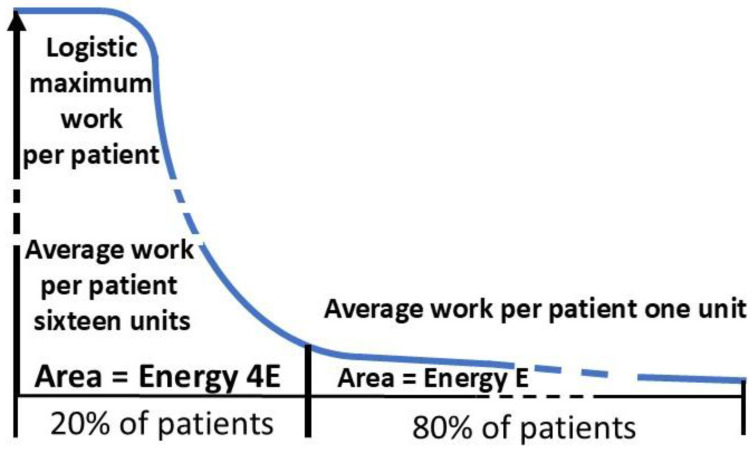
The likely distribution of work per patient for Pareto 80/20.

## Data Availability

No new data were created or analyzed in this study.

## Appendix A. Accuracy of the Likelihood Ratio in Calculating P(ASD|MI)

The HR method has interesting properties. In deriving the formula, the term HR will be used, but the approximation of the LR by using old, biased values will also apply to the RR. There is an extra approximation for the HR alone, which will be discussed after the formula is derived. The HR is mathematically P(MI|ASD)/P(MI|not ASD). The numerator P(MI|ASD) is itself a ratio of the measured MI patients who are comorbid with the measured ASD. If the ratio is the same, whatever the number of ASD patients found, i.e., P(MI|ASD) is constant, then the number of ASD patients identified does not matter. ASD presents earlier than MI (Section 3.1.1), and by the time MI is reliably diagnosable there will be a sufficient base of ASD to form the denominator of P(MI|ASD). The HR denominator P(MI|not ASD) consists of the balance of the MI patients who are not comorbid with ASD. There are three MI groups, i.e., those comorbid with ASD and measured as such, those comorbid with ASD which has not itself been measured, and those who are not associated with ASD. Since the hazard ratio is a ratio of the MI groups MI|ASD and MI|not ASD, any fractional error in MI measurement will cancel as long as it is fairly constant across the three groups. We do not actually need to know the true P(MI). Changes in the measured value of P(ASD) do affect the value of P(MI|ASD)/P(MI|not ASD). The value does not vary much over a wide range of the relationship between ASD and MI, however, and generally underestimates HR by a small margin. The estimates of P(ASD|MI) are then conservative. We will now derive a formula to find P(ASD|MI) from the HR.

The Venn diagram in Figure 3 can be transformed into a Bayesian 2 × 2 table. The 2 × 2 table, or Carrollian Table, was invented by Charles Lutwidge Dodgson, *nom de plume* Lewis Carroll, who was a mathematics don at Oxford University. He was a logician who introduced the 2 × 2 table as the *biliteral diagram* in 1896 [152].

In Figure A1, the quadrants correspond to the areas in the Venn diagram as follows:

MI is being used as a test for ASD, and the likelihood ratio is mathematically equivalent to the hazard ratio, i.e., P(MI|ASD)/P(MI|not ASD). P(ASD|MI) is the positive predictive value, the probability that ASD is present given that MI is present, i.e., a “positive test.”

We will first derive the general LR formula as follows:

Let P(ASD) be *A* and its fractional error be *f.* Then the measured P(ASD) is *Af*. Let P(MI) be *M* and its fractional error be *g*. Then the measured P(MI) will be *Mg*. Let P(MI|ASD) be *K*. Our measured P(MI|ASD) will be *Kg* since the error *g* is assumed to be constant for both *M* and *K*. The fractional error *f* is set at 1.0 for a P(ASD) of 0.060 [18]. We construct the 2 × 2 table, as seen in Figure A2, using probabilities:

Then to find LR, i.e., (a/(a + *c*))/(*b*/(*b* + *d*))

*a*/(*a* + *c*) = *AfKg*/*Af*

=*Kg*

*b*/(*b* + *d*) = (*Mg* − *AfKg*)/(1 − *Af*) (A1)

*LR* = *Kg*(1 − *Af*)/(*Mg* − *AfKg*)

=(*K* − *AfK*)/(*M* − *AfK*)

Given our assumption of a constant fractional error in MI measurement, the *g* cancels, so we do not have to know the true *K* or *M*. Since *Af* has been thought to be 0.01 or less in the past and *AfK* must be even smaller since K < 1, the past values of the HR are not much different to (*K* − *AK*)/(*M* − *AK*), which gives the contemporary LR for calculating P(MI|ASD). Since *f* in past measurements has always been <1 due to underestimating P(ASD), the denominator *M* − *AfK* has always been larger than *M* − *AK*, since *AfK* < *AK*. In the range of conditions we are dealing with, *M* < *K*. This leads to (*K* − *AfK*)/(*M* − *AfK*) < (*K* − *AK*)/(*M* − *AK*), which produces a reduction in LR compared to the true value. Then past measurements of HR produce a lower LR, and therefore a more conservative P(ASD|MI).

We will now look at some realistic examples. P(ASD|MI) is usually in about the range of 0.15–0.30 (Table 2), P(MI) usually ranges from about 0.02 (AN) to 0.12 (PTSD, PND), P(MI|ASD) up to 0.60 (PND), and P(ASD) from 0.01 to 0.06. For ease of understanding in the 2 × 2 tables, we will consider a female population of 1000 representing a probability of one.

In Figure A3, 2 × 2 Figure A3A uses values of 0.060 (=60 patients) for P(ASD) and 0.12 for PND (=120 patients). In 2 × 2 Figure A3B, we measure only half the PND patients in both squares a and b (Figure A1), and the LR does not change.

In Figure A4, 2 × 2 Figure A4A is the same as Figure A3A. In 2 × 2 Figure A4B, we reduce P(ASD) from 0.060 to the current biased value of 0.010. This reduces the value of the LR and translates to a reduction in predicted P(ASD|PND) from 0.30 to 0.25, or 16.7%. In this sequence of calculations, P(PND) was much larger than P(ASD).

Figure A5 shows the situation for anorexia nervosa when the P(AN) of 0.02 is 1/6 the value of P(PND) and 1/3 the value of unbiased P(ASD) [18]. The generally accepted range of estimates of P(ASD|AN) is from 0.20 to 0.30 [75]. The value for P(ASD|AN) from 2 × 2 Figure A5A is 0.250, and 2 × 2 Figure A5B gives 0.216. The reduction in P(ASD|AN) is 13.6%. This exercise demonstrates that the error over a wide range of MI/ASD relationships calculated using the HR is not likely to be clinically significant.

Then, for calculating a contemporary value for P(ASD|MI) using the HR as LR and the P(ASD) of 0.060,

Odds(ASD) = 0.060/(1 − 0.060)

=0.0638

Odds(ASD|MI) = LR × Odds(ASD)

=*F_A_*_1_ × 0.0638 (A2)

P(ASD|MI) = 0.0638*F_A_*_1_/(1 + 0.0638*F_A_*_1_)

=*F_A_*_1_/(15.667 + *F_A_*_1_)

=*HR*/(15.667 + *HR*)

The relative risk (RR) gives an underestimate of the LR for the following reason: females with ASD are the at-risk subset of the population. Then, the risk of MI is P(MI|ASD). For the balance of the population, the risk of MI is P(MI|not ASD). Then, the relative risk is

P(MI|ASD)/P(MI/not ASD)

which is the formula for the LR, but the true relative size of the populations ASD and not ASD are skewed by the error in the size of P(ASD), as was demonstrated above. Extended to the HR, an HR compares the instantaneous event rates (hazards) of two populations as time-to-event data [153]. Under the proportional hazards assumption, if events are rare, HR approximates RR and the simplified RR = HR formulation can be used. The rare events assumption applies to the event (MI), not to the exposure (ASD). The event (MI) should be uncommon in the baseline population (not ASD). The secondary sources giving n HR assume proportional hazards. The acceptable baseline prevalence of the risk is up to about 0.15 (15%). Data for anxiety and depression in children and adolescents vary widely, but both conditions probably qualify. It must be noted that higher values actually produce a lower RR approximation, so the values for P(ASD|MI) in this paper using n HR are almost certainly an overall underestimation. The purpose of this study has been to show that the magnitude of P(ASD|MI) estimations are high enough to require routine investigation, and this has been demonstrated.

## Appendix B. Degree of Benefit: Pareto Calculations

### Appendix B.1. The Pareto Principle in Health

The Pareto principle is commonly called the 80/20 rule, where 20% of the cause leads to 80% of the effect. While the variable strength of a particular cause will be a continuous power function, the division into two parts comparing the effect of the average of the strongest 20% of the cause to the average of the remaining 80% is a useful simplification. There may be multiple causes depending on the scenario, but for the effect of ASD on female mental health, we will consider ASD as an independent cause. From Table 2 it can be seen that the prevalence of ASD in most MIs is in the range of 15–30%. This makes a Pareto analysis potentially useful, since there is considerable evidence that the presence of ASD makes the MI more severe, for example, with the stress of camouflaging in depression [154,155] and with AN being more difficult and expensive to treat [Appendix B.3]. In mental health, financial cost is a useful measure but is not necessarily the limiting variable. Instead, the limiting variable is often a lack of trained staff, poorly distributed in the population. A useful analogy for the drivers is the first law of thermodynamics. The amount of energy remains constant but can be moved around to do work. We will define a unit of energy as what is necessary to do the work to deliver a unit of service to an average patient in the less-difficult group. This could equate to funding or performing a service. The two proportions do not have to be 80/20 and do not even have to add up to 100%. The Pareto principle seems to have wide application. We will now analyze why this might be so and apply it to health management.

### Appendix B.2. Derivation of Pareto Formulae

The aim is to derive formulae to estimate the gain in system efficiency of management by treating the ASD of comorbid mental health patients in a manner empathetic to neurodivergence.

To examine the general case of the relation between two causal proportions in a given MI, we will assume a binary risk with the higher-risk group having the stronger effect. Let the higher patient proportion with the weaker average effect be p and the lower patient proportion with the stronger average effect be q. Proportion p of the patients requires proportion *q* of the energy, so an average patient in group p uses *q*/*p* energy. An average patient in group q uses proportion *p*/*q* energy. Then, the energy–cost ratio of the average high-work patient in group q to the average low-work patient in group p is (*p*/*q*)/(*q*/*p*) or (*p*/*q*)^2^. For the 80/20 rule, the relative effect will be 16/1 (Figure A6).

The likely patient–work distribution for a ratio of 80/20 is shown in Figure A7.

The Pareto principle is said to be an empirical rule, but in biology, there is a plausible reason why it is rarely higher, say 90/10. Due to the exponential nature of cause and effect for the delivery of a costly labor-intensive biological service, there will be a logistical upper limit to the ratio. The absolute ceiling will be the most intensive inpatient care possible or its clinical equivalent in the system of interest. There is evidence that limited medical services do obey the 80/20 ratio [54,156,157,158], and the *p*^2^/*q*^2^ ratio of 16/1 for the difficult 20% to the easier 80% reflects the logistic ceiling.

Focusing on the needs of the higher-risk group is intuitively sensible but can be seen as inequitable if large numbers of patients receive no care at all. Comorbid ASD is going to have a strong effect on the MI. We will quantify how diagnosing the comorbid ASD leads to a dramatic improvement in the entire group with the MI.

If we assume the energy cost of the service is the sum of the costs of individual consultations, then the energy cost of the visits of the high-work group q is as follows:

*q* × *p*^2^/*q*^2^

*p*^2^/*q*

The energy cost of the visits of the low-work group p is *p* × 1.

The total energy cost in groups p and q in units of the work per average patient in group p is as follows:

*p*^2^/*q* + *p*

(*p*^2^ + *pq*)/*q*

*p*(*p* + *q*)/*q* (A3)

In a typical current, reasonably well-functioning health system, the limiting variable will be staff availability. Group q will have been triaged for treatment, but there will be a long waiting list for inclusion in the less-severely affected group p. Since ASD makes MI more difficult to treat, the comorbid patients will be distributed preferentially into the higher-work group q. In calculating a formula for the distribution of the comorbid ASD patients in the entire (p + q) group of patients with a mental illness, we will assume that, on average, the patients without ASD in each group, p and q, will require the same range of energy input as those with ASD. With an overall P(ASD|MI) of nearly 20%, it is likely that most of the patients in group q will have ASD. ASD should, however, be sought when assessing all MI patients since the patients in group p will also benefit. ASD does have continuous traits [159], which will be distributed over a number of further patients in both groups. These patients will also benefit from an empathetic approach to neurodivergence and contribute an extra unmeasured benefit to the final outcome.

We then start with (p + q) patients with a mental illness (MI).

The average group q patient needs *p*^2^/*q*^2^ units of work. The average group p patient needs one unit of work.

Let the proportion of P(ASD|MI) with comorbid ASD be *m*.

Then the number of patients with ASD is *m*(*p* + *q*).

Let the proportions of patients with ASD in groups p and q be *s*/(*r* + *s*) and *r*/(*r* + *s*).

Then the total work required by ASD patients in group q is *p*^2^/*q*^2^ × *m*(*p* + *q*) × *r*/(*r* + *s*).

The total work required by ASD patients in group p is 1 × *m*(*p* + *q*) × *s*/(*r* + *s*).

The total work required for all comorbid ASD patients is as follows:

*p*^2^/*q*^2^ × *m*(*p* + *q*) × *r*/(*r* + *s*) + 1 × *m*(*p* + *q*) × *s*/(*r* + *s*)

*m*(*p* +*q*) (*p*^2^*r* + *q*^2^*s*)/*q*^2^(*r* + *s*)

Let the average fractional efficiency gain by treating ASD patients in an empathetic neurodivergent manner be *e*. Then the total reduction in work needed (=energy saved) in units required to treat group p patients is as follows:

*em*(*p* +*q*) (*p*^2^*r* + *q*^2^*s*)/*q*^2^(*r* + *s*) (A4)

This energy can be used to service an extra *em*(*p* +*q*) (*p*^2^*r* + *q*^2^*s*)/*q*^2^(*r* + *s*) patients in the low-work category p, providing that there is no removal of energy from the system. The rule should be a guide to the redistribution of resources, not a way to reduce them. Identifying ASD, especially in group q, gives a considerable positive opportunity dividend for therapy, where the clinical need far exceeds the resources. This is not immediately apparent from the formula, and we will calculate a plausible scenario.

### Appendix B.3. A Worked Example

We can use data from anorexia nervosa to derive an indicative overall energy benefit. The 80/20 rule does appear to apply approximately for AN. About 20% of patients are refractory to treatment [160]. P(ASD|AN) is about 25% [18]. It is known that this group is more difficult to treat than groups with AN alone [11,12,13,50,161]. Successful treatments for this group are being found: a study [125] has shown that managing diagnosed inpatient AN/ASD patients accounting for their neurodivergence is effective and saves 32.2% in costs per patient. Then an indicative *e* is 0.322. P(ASD|AN) gives *m* as 0.25. Most comorbid patients are likely to be in Pareto group q. A reasonable starting point, then, for an indicative efficiency dividend would be with both *p*/*q* and *r*/*s* being 80/20.

Then, the energy saved in group p patient equivalents by Formula (A4) is as follows:

0.322 × 0.25 × 100(80^3^ + 20^3^)/20^2^(100) = 83.72

We have more than doubled the number of low-work patients treatable in female AN, where a proportion is comorbid with ASD, by recognizing the ASD and managing it appropriately. This is the substantial result of diagnosing a common unrecognized but treatable comorbidity (ASD) which caused significant management problems in the initial diagnosed mental illness.

This example demonstrates how the study stimulates useful hypotheses. When we know P(ASD|MI) is clinically significant, we can ask the following question: what proportion of female patients with difficult-to-treat MIs have comorbid ASD and what are the funding implications? “Difficult” would, of course, need to be defined, and the mental illness designated. An observational study could follow and be compared with the theoretical result obtained above.

### Appendix B.4. Downstream Effects

If a treatment gain is achieved, there will be a major positive ripple effect due to significant improvements in personal, family, and societal problems associated with unrecognized and undertreated mental illness. The ratio of direct treatment cost-to-overall societal cost for mental illness has been estimated to be about 4.7 [162]. We then find that the total useful societal energy gain (“heat” loss foregone) for our AN example is as follows:

4.7*em*(*p* + *q*) (*p*^2^*r* + *q*^2^*s*)/*q*^2^(*r* + *s*)

For every 100 patients (*p* + *q*), we would divert enough energy to treat an extra 393.5 group p patients, though in practice most of the energy would be potential, appropriately preventing adverse consequences in the downstream community.

As an example of downstream savings, Luca [65] found the average cost of an untreated postnatal depression mother/child dyad from conception to 5-years postpartum was USD 31,800. P(ASD|PND) from Section 3.1.3 is 0.313, so 31.3% of these dyads would be complicated by ASD. The societal ripple effect would give an extended average cost of 4.7 × 31,800 or USD 149,460 per dyad. An example of a useful intervention would be the increased diagnosis of neurodevelopmental problems in the offspring [46]. We can calculate the overall savings achieved by screening for maternal ASD in the antenatal period. Assume the costs of no treatment have a Pareto distribution and let the low initial average cost for the mother/child dyad in group p over 5 years be *z*. The total cost of the patients (*p* + *q*) by Formula (A3) is as follows:

*zp*(*p* + *q*)/*q*

The average dyad cost including ripple effect over 5 years is as follows:

*zp*(*p* + *q*)/*q*(*p* + *q*)

*zp*/*q*

*zp*/*q* = 149,460

The proportion of patients with ASD is high, at 31.3%. A realistic Pareto distribution would be *p*/*q* of 70/30. Then the low average extended cost *z* is USD 64,054. The therapeutic effect of a diagnosis for the mother is probably going to be quite high. The child would also be assessed as having an increased probability of ASD, and, if diagnosed, would receive early intervention, so aiming for a 50% savings (*e* = 0.5) for comorbid dyads would probably be conservative. The proportion of ASD patients in group q is likely to be very high, say *r*/*s* in groups q and p, respectively, of 90/10. Then, by Formula (A4), the cost saving as multiples of *z* is as follows:

0.5 × 0.313 × 100(70^2^ × 90 + 30^2^ × 10)/(30^2^ × 100)

76.685

Then, the total 5-year extended cost saving is USD 64,054 × 76.685, i.e., USD 4,911,981 for every (*p* + *q*) patients, giving the % savings as follows:

100 × 4,911,981/14,946,000

32.9%

USD 49,120 per dyad.

The substantial overall saving across all dyads is because most of the ASD/PND dyads are in the high-cost Pareto group q. A 2023 paper on new screening recommendations for PND [163] describes PND as the leading cause of overall and preventable maternal mortality. This will obviously cause severe downstream effects. The paper listed several comorbid mental illnesses but did not mention autism. A 2024 study [110] using Swedish National Registry data examined the aftermath of perinatal depression (PeND) from 2001 to 2017, inclusive. They observed that suicide from PeND accounts for 20% of maternal deaths in high-income countries. Women with postpartum psychiatric disorders, including psychotic, affective, and anxiety disorders, have an increased risk of death. They found that women with PeND had a high risk of death, closely after diagnosis with a hazard ratio of 12.17 for suicide. This was attenuated over 12 months postpartum but was still elevated (HR~2) 18 years later, compared to their parous genetic sisters who did not have PeND. This was independent of a pre-pregnancy psychiatric diagnosis and was more common under 30 years of age. The cause was unclear. They concluded that PeND required urgent detection and treatment. The possibility of comorbid ASD was not discussed, but from their data, P(ASD|PND) may have been up to 40%. There are physical risks for autistic mothers and their babies, including pre-term delivery, especially extreme pre-term, small for gestational age [164], increased ceserean delivery, and preeclampsia [165], all of which may increase postnatal stress. The practical outcome of these results is the need to redesign a significant proportion of female mental health diagnosis and management.

## Appendix C. Calculate P(ASD) from the Value of P(ASD|CS) of 0.415 [92]

Let the relative risk L remain constant at 10.667 (Section 3.2.2), the P(ASD|CS) be S, the new P(ASD) be A, and the odds of the new ASD be O(ASD). Then

O(ASD) = *A*/(1 − *A*)

O(ASD|CS) = *L* × *A*/(1 − *A*)

*S* = (*LA*/(1 − *A*))/(1 + *LA*/(1 − *A*))

= (*LA*/(1 − *A*))/(1 − *A* + *LA*)/(1 − *A*)

= *LA*/(1 − *A* + *LA*)

Then

0.415 = 10.667*A*/((1 − *A*) + 10.667*A*)

0.415 − 0.415*A* + 0.415 × 10.667*A* = 10.667*A*

*A*(10.667 + 0.415 *−* 4.427) = 0.415

*A* = 0.415/6.665

P(ASD) = 0.062

## Appendix D. The Risk of Autism with Exposure to Acetaminophen in Pregnancy

The study with the most rigorous statistical methodology was a very large study [166] using Swedish health registers and included 2,480,797 children, with 185,909 exposed to acetaminophen. After sibling analysis to minimize confounding variables, the hazard ratio for autism was 0.98. Since it is unlikely that acetaminophen prevented autism, we will use a HR of 1.0 in Formula (2):

HR/(15.667 + HR)

1/(15.667 + 1)

0.060

The formula correctly gives the base population prevalence of female ASD. Acetaminophen in pregnancy does not cause an increase in autism.
